# Engineering of an *E. coli *outer membrane protein FhuA with increased channel diameter

**DOI:** 10.1186/1477-3155-9-33

**Published:** 2011-08-19

**Authors:** Manuel Krewinkel, Tamara Dworeck, Marco Fioroni

**Affiliations:** 1 Department of Biotechnology (Biology VI), RWTH Aachen University, Worringerweg 1, 52074 Aachen, Germany

**Keywords:** Channel proteins, FhuA, liposomes, protein engineering, HRP, TMB-Assay, nanocontainers

## Abstract

**Background:**

Channel proteins like FhuA can be an alternative to artificial chemically synthesized nanopores. To reach such goals, channel proteins must be flexible enough to be modified in their geometry, *i.e*. length and diameter. As continuation of a previous study in which we addressed the lengthening of the channel, here we report the increasing of the channel diameter by genetic engineering.

**Results:**

The FhuA Δ1-159 diameter increase has been obtained by doubling the amino acid sequence of the first two N-terminal β-strands, resulting in variant FhuA Δ1-159 Exp. The total number of β-strands increased from 22 to 24 and the channel surface area is expected to increase by ~16%. The secondary structure analysis by circular dichroism (CD) spectroscopy shows a high β-sheet content, suggesting the correct folding of FhuA Δ1-159 Exp. To further prove the FhuA Δ1-159 Exp channel functionality, kinetic measurement using the HRP-TMB assay (HRP = Horse Radish Peroxidase, TMB = 3,3',5,5'-tetramethylbenzidine) were conducted. The results indicated a 17% faster diffusion kinetic for FhuA Δ1-159 Exp as compared to FhuA Δ1-159, well correlated to the expected channel surface area increase of ~16%.

**Conclusion:**

In this study using a simple "semi rational" approach the FhuA Δ1-159 diameter was enlarged. By combining the actual results with the previous ones on the FhuA Δ1-159 lengthening a new set of synthetic nanochannels with desired lengths and diameters can be produced, broadening the FhuA Δ1-159 applications. As large scale protein production is possible our approach can give a contribution to nanochannel industrial applications.

## Background

Integral outer membrane proteins of gram negative bacteria use amphiphatic β-sheets to traverse lipid membranes. β-barrel proteins consisting of 8, 12, 14, 10, 18 and 22 strands are known. All members of the above mentioned family are cylindrical, closed barrels with an even number of transmembrane β-strands that are connected in a β-meander topology with alternating tight turns and longer connecting loops [1]. The β-strand contribution to the overall secondary structure of these proteins is usually high (~ 60%) [1-5]. The respective membranes are spanned by β-strands of 9-11 residues. The smallest known barrel (*i.e*. OmpA) contains 8 transmembrane strands; due to packing constraints in the barrel interior, this might mark the lower possible size limit [2]. The largest known β-barrel proteins contain 22 strands (*i.e*. TonB dependent importers). However there is some evidence for the existence of even larger β-barrels [3]. In general the hydrophobic and membrane-interacting surface of β-barrel proteins is cryptically encoded in their primary sequence [4].

Apart from their biological importance, one application of bacterial membrane proteins with β-barrel structure is the channel functionalization of lipid or block copolymer based membranes. So far the bacterial nucleoside transporter Tsx, which is one of the smaller β-barrel proteins with 12 antiparallel strands [5], the *E. coli *outer membrane protein F (OmpF), with 16 antiparallel β-strands [6], the *E. coli *mechanosensitive channel protein MscL and one of the largest β-barrel proteins, the *E. coli *Ferric hydroxamate protein uptake component A (FhuA) have been successfully inserted into lipid or polymer based vesicles [7-9]. Especially the FhuA proved to be useful, due to its wide channel diameter and robustness against for instance tryptic digestion [10].

The *E. coli *outer membrane protein FhuA is one of the largest known β-barrel proteins (714 amino acids, elliptical cross section 39*46 Å), consisting of 22 antiparallel β-strands connected by short periplasmatic turns and flexible external loops. The protein channel is closed by a cork domain (amino acids 5-159). Several crystal structures of the FhuA wild type have been resolved [11,12]. The number of amino acids spanning the outer membrane is 9 to 10 for each β-strand [12]. For biotechnological applications one FhuA variant has been engineered in which the cork domain has been removed (FhuA **Δ**1-159, *i.e*. deletion of amino acids 1 - 159), resulting in a passive mass transfer channel [13]. The FhuA Δ1-159 variant has been inserted as a nanochannel triggered by chemical external stimuli into PMOXA-PDMS-PMOXA [PMOXA - poly(2-methyl-2-oxazoline); PDMS - poly(dimethyl-siloxane)] triblock copolymer membranes [14] or liposomes respectively [15]. Very recently FhuA Δ1-159 was specifically altered to insert it into thick membranes formed by cheap triblock copolymer PIB_1000_-PEG_6000_-PIB_1000 _(PIB = poly-isobutylene, PEG = poly-ethylen-glycol). For this purpose the protein hydrophobic region was elongated from 3 to 4 nm by "copy-pasting" the last five amino acids of each β-sheet on the periplasmatic side of the barrel [16].

Previous findings show that FhuA and its variants are applicable as nanochannels in liposome or polymersome systems. Furthermore engineered FhuA variants can be an alternative to nanochannels based on artificial β-barrel structures able to insert into the lipid bilayer [17-19]. These artificial and self-assembling β-barrels are based on rigid rod molecules - extremely rigid, synthetic rod-shaped molecules with great potential in material sciences - combined with short peptide strands. Artificial β-barrels can in principle be tailored in size and functional groups [17,20]. However the synthesis of these molecules is not trivial and large scale production is difficult [17]. Furthermore the peptide sequence variety is until now very limited and each artificial β-barrel contains only one kind of peptide sequence [21,22].

To overcome the limitations in synthesis and related scale up, the use of β-barrel proteins modified by genetic engineering techniques can be considered a valid alternative.

Here we report for the first time the successful re-engineering of a channel protein with β-barrel structure leading to an increase in channel diameter. The channel diameter of FhuA variant Δ1-159 was increased by addition of two β-strands to the protein primary sequence. As the folding information is intrinsically contained in the primary sequence, it seemed most promising to copy a part of the already existing sequence, therefore the first N-terminal β-sheet was doubled to reach FhuA variant Δ1-159 Exp (expanded diameter). The FhuA Δ1-159 Exp secondary structure was analyzed by circular dichroism (CD). To demonstrate the functionality of FhuA Δ1-159 Exp as a channel, kinetics for TMB (3,3',5,5'-tetramethylbenzidine) uptake by HRP (Horse Radish Peroxidase) loaded liposomes with inserted open and biotin label-closed FhuA Δ1-159 Exp channel were measured. The kinetic data were compared to results of identically carried out experiments with FhuA Δ1-159.

## Results and Discussion

### FhuA Δ1-159 Exp conceptual and estimated increase in diameter

**Figure 1:**
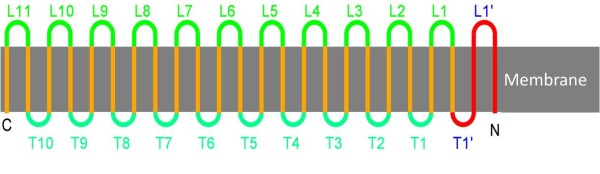
**Schematic representation of FhuA Δ1-159 Exp design**. Schematic picture of FhuA **Δ **1-159 Exp secondary structure as it transverses the membrane (depicted in grey). Membrane spanning **β**-strands are marked in orange, outside loops (L1' - L11) in green and periplasmic turns (T1' - T11) in turquoise. The two N-terminal doubled β-strands as well as the associated turns and loops are depicted in red.

The aim was to increase the inner channel diameter of *E. coli *outer membrane protein FhuA Δ1-159, by the addition of a further β-sheet. A conservative approach was chosen, starting with the addition of only two strands, without introducing entirely new sequence information, but rather by copy-pasting 30 amino acids of the existing sequence. The first two strands on the N-terminus were chosen (Figure 1), as they are connected by one of the shortest loops. The N-terminal sequence is preserved and thus N- and C-terminus are still expected to close to the intact barrel structure. Furthermore the chosen β-stands are rather suited for duplicating, as they are composed 61% of amino acids that promote β-sheet formation [23].

**Figure 2:**
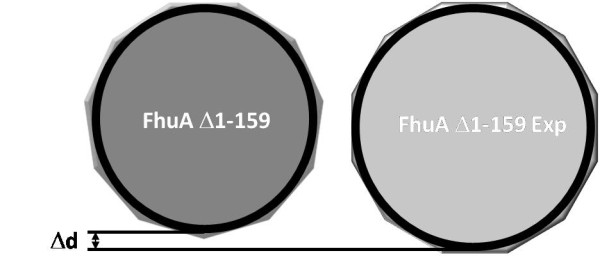
**Comparison of FhuA Δ1-159 and FhuA Δ1-159 Exp channel diameter**. FhuA Δ1-159 is represented as a docosagon and FhuA Δ1-159 Exp as a tetracosagon. The side length is constrained due to the constrained hydrogen bond length. Increasing the number of β-strands from 22 (FhuA Δ1-159) to 24 (FhuA Δ1-159 Exp) leads to an increased diameter, as indicated (Δ d).

Since the FhuA shows a nearly circular morphology [11,12], a simple regular polygonal geometry (hendecagon for FhuA Δ1-159 and dodecagon for FhuA Δ1-159 Exp), with constrained side length given by the hydrogen bond connecting the β-sheets, can be assumed (Figure 2). Based on this assumption the relative expected diameter increase can be calculated, as the FhuA wild type (WT) channel diameter with 22 β-sheets is known to be ~4.2 nm as deduced from crystal structure [11,12]. Knowing the apothem of FhuA WT, the expected FhuA Δ1-159 Exp pore cross section is ~4.6 nm as calculated from the apothem ratio. Thus the channel surface area increases by 16% upon addition of two β-strands (see also Additional File 1 Table S1).

### Extraction and Purification of FhuA Δ1-159 Exp

**Figure 3:**
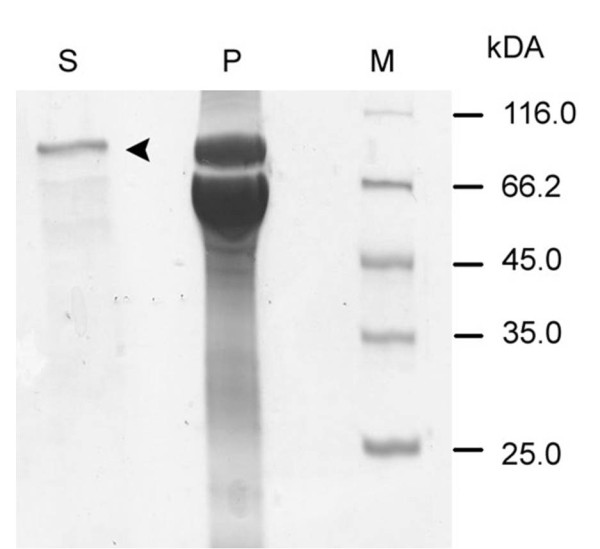
**SDS-PAGE result of FhuA Δ1-159 Exp extraction (using detergent OES)**. S - sample of FhuA Δ1-159 Exp in OES (expected size: ~66 kDa). P -lipid fraction pellet remaining after protein extraction. M - protein molecular weight marker (Fermentas, St. Leon-Rot, Germany).

Detergent extraction led to FhuA Δ1-159 Exp solubilized in buffer containing detergent oPOE (n-octylpolyoxyethylene). The sample contained many impurities (whole protein content: ~1800 μg/ml) and was therefore discarded. Second extraction step using the detergent OES (n-octyl-2-hydroxyethylsulfoxide) led to ~420 μg/ml of FhuA Δ1-159 Exp (Figure 3). ImageJ (http://rsbweb.nih.gov/ij/index.html) analysis resulted in a FhuA Δ1-159 Exp purity of ~80-90% for the OES solubilised sample. The ImageJ program converts photographic plates (analogic) to a digitalized matrix where to each pixel (x axis) is assigned a value, 0 for white and 1 for black passing through a grey scale (y axis). The total area or number of pixels with values > 0 corresponds to the protein bands on a SDS gel within one lane and is normalized to 100%. At this point the relative purity of a protein in the same lane is simply deduced by determining the ratio between the number of pixels > 0 corresponding to the interesting protein band and the number of pixels > 0 of all bands in the lane.

### CD Spectra of FhuA Δ1-159 Exp

Based on the observation that the original FhuA Δ1-159 is able to independently refold after thermal denaturation (unpublished data from the authors) or when extracted from inclusion bodies [24], showing that folding information is fully contained within the primary sequence [4], the FhuA Δ1-159 Exp was expected to fold as a β-barrel.

The secondary structure analysis of FhuA Δ1-159 Exp by circular dichroism (CD) gives clues on whether the applied engineering strategy to widen the inner protein channel diameter leads still to a β-sheet folding.

**Figure 4:**
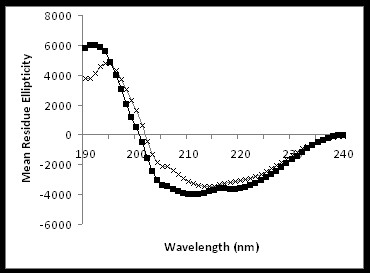
**FhuA Δ1-159 Exp CD spectrum and fit**. Spectrum was obtained in a potassium phosphate solution (0.1 M, pH 7.4) containing 0.5% (w/v) of OES detergent.

In agreement with previous CD results obtained for WT, FhuA Δ1-159 as well as WT crystal structure [11], the secondary structure of FhuA Δ1-159 Exp is well retained as shown in Figure 4.

The CD derived amount of β-structure for FhuA WT is 51% [14], while for FhuA Δ1-159 values between 49% and 65% have been reported [24,25].

The deconvolved dichroic spectra using the CONTIN [26] method report a 63% of β-strand, 30% random coil and 7% α-helical contribution for the FhuA Δ1-159 Exp (data fitting and errors are shown in Additional file 2) and 57% of β-strand, 31% random coil and 12% α-helical contribution for FhuA Δ1-159 (Figure 4) [24].

Though the secondary structure shows the expected trend, the CD technique cannot provide information on a protein tertiary structure thus the β-barrel closing cannot definitely be proved. In the following section kinetic studies on liposome inserted FhuA Δ1-159 Exp address the channel functionality and suggest a β-barrel folding.

### Influx kinetics and TMB/HRP detection system

To verify whether FhuA Δ1-159 Exp is functional, the protein was reconstituted into liposomes made of *E. coli *lipid extract. The channel functionality was analysed with the help of the HRP/TMB assay system. This method was already applied in order to study the channel properties of membrane isolated FhuA Δ1-159 [15].

The TMB/HRP is a widely used enzymatic assay, its detection system is based on a two-step irreversible consecutive reaction A→B→C (A = TMB; B and C = first and second TMB oxidation products) catalysed by enzyme HRP in presence of H_2_O_2_. Since the final TMB oxidation product C is only stable under very acidic conditions (0.3 Mol/L H_2_SO_4_) [27], the intermediate product (B) is used as a reporter with a characteristic absorbance maximum at 370 and 652 nm. TMB oxidation kinetics were quantified by measuring absorption at 652 nm over a time of 9 min.

The HRP was encapsulated into liposomes and despite of using the Soret absorption band, the total amount of encapsulated enzyme could not be detected.

The kinetic data obtained in presence of the FhuA Δ1-159 Exp, were compared to a negative control consisting of HRP loaded liposomes, to verify the obtained results. Empty liposomes were used as a blank as they show no kinetics and their self-absorption was subtracted from all kinetic data.

**Figure 5:**
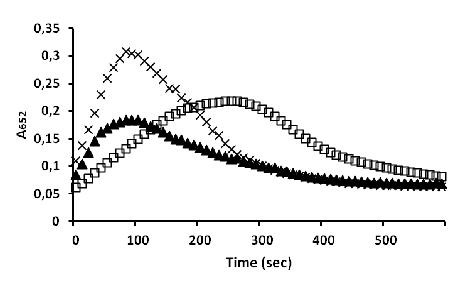
**Results of TMB conversion kinetics**. HRP loaded liposomes (squares), HRP loaded liposomes with reconstituted FhuA Δ1-159 Exp (crosses) and HRP loaded liposomes with label blocked FhuA Δ1-159 Exp (triangles).

Overall kinetic data are reported in Figure 5.

Since the lipid membane is not entirely impermeable, liposomes lacking the channel protein show slow TMB conversion kinetics (Figure 4, squares), however TMB conversion of lipid vesicles with inserted FhuA Δ1-159 Exp (Figure 4, crosses) occurs ~11-times faster. A linear regression using "least square" method was performed to find the best linear section in the steepest region of both curves, to determine the absorbance time derivative (see details in Additional File 1, Figure S2). Lambert Beer Law was used to calculate the TMB conversion speed in nM/sec (Table 1) [24]. To address the question whether the FhuA Δ1-159 Exp truly acts as a channel or whether the liposome membrane gets locally perturbed by the presence of the protein, FhuA Δ1-159 Exp was blocked by biotinylation of the 31 Lys-NH_2 _groups, using a technique that has already been applied to block the FhuA Δ1-159 [14,15] and FhuA Δ1-159 Ext [16] (see next paragraph).

**Table 1:** Comparison of FhuA Δ1-159 and FhuA Δ1-159 Exp (unblocked and blocked) average TMB conversions in liposomes

Sample	TMB conversion [nM]/sec	Study
Liposomes + HRP + FhuA Δ1-159 Exp	162 ± 3	This study

Liposomes + HRP + FhuA Δ1-159 Exp (blocked)	54 ± 2	This study

Liposomes + HRP + FhuA Δ1-159	139 ± 7	[24]

Liposomes + HRP + FhuA Δ1-159 (blocked)	14 ± 1	[24]

The previous experiments showed the ability of channel Lys-NH_2 _group biotinylation to efficiently close the channel in case of FhuA Δ1-159 (means no detectable substrate conversion) or to at least a partial closing of the channel in case of the elongated FhuA Δ1-159 Ext (means drastical decrease of substrate conversion speed).

In comparison to liposomes harbouring the open FhuA Δ1-159 Exp, liposomes with the blocked channel protein show a ~3 times smaller slope and TMB conversion rate (Figure 4, black triangles) (see Additional File 1, Figure S2). The incomplete channel blocking can be most likely explained by the expected increase in channel diameter. As the two extra Lys-NH_2 _groups that were introduced by the addition of two β-strands should face the channel exterior (as in the original β-strands in FhuA Δ1-159), labelling of these two functional groups can therefore give no contribution to the blocking. The possibility that not all 31 Lys-NH_2 _groups were labelled could be excluded by determining the overall amount of biotin labels present on the protein (as described in the next section: "Quantitative determination of the biotinylated Lys") and comparing to expected theoretical amount of labels.

A comparison of FhuAΔ1-159 Exp kinetic data and data obtained for FhuA Δ1-159 as derived from a previous study [24] is shown in Table 1. It shows that TMB conversion and therefore TMB translocation through the liposomes inserted FhuA Δ1-159 Exp occurs ~1.2-times (17%) faster as compared to FhuA Δ1-159, this can be correlated to the expected channel surface area increase of 16% (see Figure 2 and Additional File 1, Table S1).

In fact due to the first Fick's law, the total flux is proportional to the diffusion coefficient and the concentration gradient. As both variables were controlled by the experimental conditions maintaining the same values via the substrate concentration, the ratio (17%) between the two fluxes measured for the FhuA Δ1-159 and FhuA Δ1-159 Exp are only proportional to the surface areas of the two channel with different diameters (16% higher for FhuA Δ1-159 Exp).

TMB conversion through the biotinylated FhuA Δ1-159 Exp takes place ~3.7-times faster than in case of the labelled FhuA Δ1-159, showing ones more that the increase in inner channel diameter leads to less efficient closing of FhuA Δ1-159 Exp.

The observed increase in diffusion kinetics through FhuA Δ1-159 Exp as compared to FhuA Δ1-159 strongly suggest that the addition of the two β-strands led to a diameter increase thus implying the correct folding of added β-strands.

This conclusion is based on the assumption that the number of channel proteins per liposome (FhuA Δ1-159 and FhuA Δ1-159 Exp) are equal. Unfortunately we have not been able to demonstrate this equality in the number.

Therefore the good correlation between channel surface area increase and kinetic results can be either due to an increase in channel diameter or to an unlikely increase in the number of inserted protein channels of exactly 16%.

### Quantitative determination of the biotinylated Lys (biotinylation assay)

Determination of the number of effectively labelled Lys-NH_2 _groups present in the FhuA Δ1-159 Exp gives a clue on whether the differences in FhuA Δ1-159 Exp influx kinetics are due to insufficient labelling or rather and as expected to the diameter increase. FhuA Δ1-159 Exp contains 31 Lys residues in total. An average biotin concentration of ~4000 pmol was found after proteolytic digestion of the labelled FhuA Δ1-159 Exp (exposing all biotin moieties), matching the theoretical biotin label concentration of 4185 pmol in case all 31 Lys are labelled (see paragraph "Biotinylation Assay" in Additional File 1). Therefore the residual kinetics cannot be accounted to a partial FhuA Δ1-159 Exp labelling.

## Conclusions

Recently many efforts have been devoted to obtain synthetic pores with β-barrel conformation having vast technological application ranging from drug-release, host sensors and catalysis [18]. These artificial β-barrels can in principle be tailored in size and functional groups [17,20]. An alternative strategy involves the engineering of bacterial β-barrel proteins that can be altered in the geometry (diameter, length) and functional groups. A first step in the direction of biological β-barrels with tailored geometry is the previously reported increase of the FhuA Δ1-159 length by 1 nm [16].

In this study in order to complete the channel geometry engineering, the FhuA Δ1-159 area was increased by 16% by increasing the number of β-strands from 22 to 24. To our knowledge this is the first time, besides our previous study [16], a channel protein was specifically engineered to modify its geometry.

A simple "copy-paste" strategy applied to the first two β-strands at the FhuA Δ1-159 N-terminus has been developed, resulting in protein variant FhuA Δ1-159 Exp (Expanded). In order to minimize the probability of incorrect protein folding a conservative approach has been followed by adding only two β-strands. The pasted amino acids are expected to lead to the same folding as the original ones, as the folding information is fully contained in the primary sequence [4]. Considering the cross section of FhuA Δ1-159 Exp as circular, the channel diameter was increased by 0.4 nm.

FhuA Δ1-159 Exp was functionally embedded into liposome membranes as confirmed by HRP/TMB assay kinetic studies. Furthermore the kinetic studies revealed an increase in diffusion kinetics of 17% for FhuA Δ1-159 Exp as compared to data obtained for FhuA Δ1-159, well in correlation with the 16% increase in total channel surface area.

The secondary structure analysis by CD spectroscopy suggests the correct β-barrel folding of the engineered FhuA Δ1-159 Exp. Overall results suggest that massive FhuA Δ1-159 engineering (addition of two β-strands) is possible without losing channel functionality.

In the future, a combination of the developed FhuA variants with increased channel length and/or diameter will give rise to a new set of synthetic channels with flexible geometry.

## Methods

All chemicals were of analytical grade or higher and purchased from Applichem (Darmstadt, Germany) or Sigma-Aldrich (St. Louis, USA). Protein concentrations were determined using the standard BCA kit (Pierce Chemical Co, Rockford, USA). The oPOE detergent was obtained from Enzo (Farmingdale, USA), while detergent OES was obtained from Bachem (Bubendorf, Switzerland).

### Construction and cloning of FhuA Δ1-159 Expanded (FhuA Δ1-159 Exp)

To increase the diameter of the FhuA Δ1-159 the first two β-sheets (30 additional amino acids in total) of the protein were copied and pasted to the N-terminus of the protein (see Figure 1).

After gene design and codon optimisation to *E. coli *using the Gene Designer software (DNA2.0 2007), the synthetic gene "fhuA Δ1-159 expanded" (fhuA Δ1-159 exp) was purchased from Mr. Gene (Regensburg, Germany). The synthetic gene (see paragraph "DNA sequence of FhuA Δ1-159 Exp", Additional File 1) was subcloned into the *E. coli *expression vector pBR-IBA1 (IBA, Göttingen, Germany) resulting in plasmid pBR-IBA1 fhuA Δ1-159 exp.

### Expression and Extraction of FhuA Δ1-159 Exp

FhuA Δ1-159 Exp was expressed in *E. coli *BE strain BL 21 (DE3) omp8 (F- hsdSB (rB- mB) gal ompT dcm (DE3) ΔlamB ompF::Tn5 ΔompA ΔompC) [29] as described previously [7] with some minor changes. Several colonies of freshly transformed *E. coli *omp8 cells were inoculated into 20 ml NaPY_Amp _medium [30]. This pre-culture was grown over night at 37°C. The main-culture was inoculated from the pre-culture to an initial OD_600 _of 0.2. Expression was performed in 200 ml NaPY_Amp _medium at 30°C. Protein expression was induced by adding 1 mM IPTG at an OD_600 _of 0.7-0.8. The expression level of FhuA Δ1-159 Exp was monitored by SDS PAGE (see section SDS PAGE). After reaching stationary phase cells were harvested by centrifugation at 3.300 × g, 4°C, 15 min and the resulting pellet was stored at -20°C until protein extraction.

FhuA Δ1-159 Exp was extracted from the *E. coli *outer membrane as previously described [31] with slight changes. All amounts are given for a pellet obtained from a 200 ml cell culture with an OD_600 _of 3.0.

The cell pellet was resuspended in 10 ml lysis buffer (0.1 M phosphate-buffer, pH 7.4; 2.5 mM MgCl_2_; 0.1 mM CaCl_2_). By the use of a high pressure homogenizator (EmulsiFlex-C3, Avestin, Ottawa, Canada) the cells were broken. Thereafter 1 ml of extraction buffer (0.1 M phosphate-buffer, pH 7.4; 2.5 mM MgCl_2_; 0.1 mM CaCl_2_; 20% v/v Triton X-100) was added and samples were incubated while shaking at 37°C for 1 hour. After incubation the solution was centrifuged at 38000 × g, 4°C for 45 min. Supernatant was discarded and the pellet was washed 3 times with washing buffer (0.1 M phosphate-buffer, pH 7.4) without resuspending it. The remaining pellet was homogenized in 1.5 ml pre-extraction buffer (0.1 M phosphate-buffer, pH 7.4; 1 mM EDTA; 0.1% v/v oPOE) using a tissue grind tube. Afterwards the solution was incubated at 37°C for 1 hour and the next centrifugation was carried out at 170000 xg, 4°C for 45 min (Beckmann Coulter Optima™, L-100-XP Ultracentrifuge, California USA). Again the pellet was resuspended using the tissue grind tube in 1.5 ml solubilisation buffer (0.1 M phosphate-buffer, pH 7.4; 1 mM EDTA; 3.0% v/v oPOE) and incubated for 1 hour at 37°C. The last centrifugation step was performed at 170000 xg, 12°C for 45 min (Beckmann Coulter Optima™, L-100-XP Ultracentrifuge, California USA) to obtain the FhuA Δ1-159 Exp protein in the supernatant. At all steps 1 μl phenylmethanesulfonyl fluorid (PMSF; protease inhibitor) was added.

From each pellet and supernatant samples were taken and analyzed on SDS gel.

To increase the FhuA Δ1-159 Exp yield, the residual pellet from the oPOE-extraction was used for further extraction with the detergent OES [32]. Therefore the pellet was resuspended in 1.5 ml OES-solubilisation buffer (0.1 M phosphate-buffer, pH 7.4; 1 mM EDTA; 0.5% w/v OES) using the tissue grind tube. After proper resuspension the solution was incubated 1 hour at 37°C with shaking. Subsequently it was centrifuged at 170000 xg, 12°C for 45 min to obtain the FhuA Δ1-159 Exp protein in the supernatant.

The purified FhuA Δ1-159 Exp was loaded onto a 10% SDS acrylamide gel [33]. After electrophoresis the protein was stained by Coomassie Brilliant blue R-250.

### SDS-PAGE

SDS polyacrylamide gel electrophoresis utilizing gels consisting of a 10% w/v acrylamide separation gel with a 5% w/v acrylamide stacking gel were used to monitor the protein expressions as well as to evaluate the quality of protein purification. Bands were visualized by Coomassie staining [33].

### Secondary structure determination of FhuA Δ1-159 Exp by circular dichroism (CD) spectroscopy

Dichroic spectra were measured with an Olis DSM 17 CD spectrophotometer (Olis, Bogard, USA). Samples were pipetted into a Suprasil QS cuvette (Hellma, Müllheim, Germany) with a pathlenght of 0.5 nm. For each samples five spectra from 190 nm to 240 nm were collected and averaged. Background spectra using the buffer, in which the protein was solved, were subtracted.

Spectra were analyzed to calculate the α-helix, β-barrel and random coil secondary structure content by using the Dicroprot deconvolution program [34] and the CONTIN algorithm [35].

### Liposome preparation

Liposomes were formed using the film hydration method [15] followed by a filter extrusion process and finally a size exclusion chromatography (SEC) for homogenization and purification.

For the lipid film preparation 500 μl of *E. coli *total lipid extract (10 mg) dissolved in chloroform (Avanti Polar Lipids, Alabasta, USA) were mixed 1:1 with methanol. The organic solvents were evaporated in a round bottom flask using a vacuum evaporator (VWR, Darmstadt, Germany) to create a lipid film in the flask. HRP (horse radish peroxidase) loaded liposomes were obtained by adding 1 ml of an aqueous buffer solution (0.1 M potassium phosphate-buffer, pH 7.4) containing HRP (2.9 U/ml) to the lipid film. Liposomes with entrapped HRP and membrane inserted FhuA Δ1-159 Exp (2.7 μM) or FhuA Δ1-159 (2.7 μM) respectively (as well as Lys-NH_2 _labeled forms of both channel proteins) were prepared by adding 1 ml buffer (0.1 M potassium phosphate-buffer, pH 7.4) containing HRP (2.9 U/ml) as well as the protein to insert (2.7 μM) to the previously formed lipid film. Empty liposomes as they were used as negative controls, were rehydrated by the addition of 1 ml buffer (0.1 M potassium phosphate-buffer, pH 7.4). Liposomes were then formed via rehydration, under slow rotation of the round bottom flasks. Lipid film preparation and rehydration were carried out over night.

Rehydrated liposomes were sequentially extruded with an Avanti polar lipids extruder (Avanti Polar Lipids, Alabasta, USA) with 1 μm, 0.4 μm and 0.2 μm membranes (Millipore, Bedford, USA) to uniform the nanocompartments in size and shape [36]. Purification was carried out through size exclusion chromatography (SEC).

### Size exclusion chromatography (SEC) for liposome purification

All five subsets of produced nanocompartments (liposomes without inserted channel protein, liposomes with inserted FhuA Δ1-159 Exp as well as liposomes with inserted FhuA Δ1-159 and the labelled forms of both channel proteins) were purified by gel filtration using Sepharose 6B (Sigma-Aldrich, Cat. no. 6B100-500 ML) in phosphate buffer (0.1 M potassium phosphate-buffer, pH 7.4).

First the SEC column was saturated with liposomes to avoid unspecific binding to the matrix. Afterwards liposomes obtained from the extrusion process were loaded. Fractions of 750 μl were collected and Average diameters of nanocompartments were routinely determined using a Zeta-Sizer (Zeta-Sizer Nano Series; Malvern, Worcestershire, UK//Supp. Mat.: Additional File 1, Figure S1).

### TMB assay with liposomes

As a reporter system for compound influx into the liposomes a colorimetric TMB conversion assay was used. Since in all types of prepared liposomes HRP is encapsulated in the lumen TMB gets converted in a two-step oxidation reaction into a blue (E = 650 nm) and subsequently yellow (E = 420 nm) product after entering the liposome.

To 50 μl of liposome solution (normalized to a OD_600 _of 0.04/ml) and 50 μl of phosphate buffer (0.1 M phosphate-buffer, pH 7.4) 20 μl of TMB-substrate solution (readymade TMB/H_2_O_2 _solution; Sigma-Aldrich, St. Louis, USA) were added. TMB oxidation kinetics was monitored at 650 nm in a Tecan Infinate M1000 spectrofluorometer (Tecan, Männedorf, Switzerland).

### FhuA Δ1-159 Exp and FhuA Δ1-159 labelling

In order to biotinylate the Lys-NH_2 _groups present on FhuA Δ1-159 Exp and FhuA Δ1-159, a 20% DMSO aqueous solution containing (2-[Biotinamido]ethylamido)-3,3'-dithiodipropionic acid *N*-hydroxysuccinimide ester) (8.2 mM) was added drop-wise to 1200 μl of a FhuA Δ1-159 Exp or FhuA Δ1-159 solution respectively and stirred (3000 rpm, 1 h; RCT basic IKAMAG, IKA-Werke GmbH, Staufen, Germany). The latter solution was used for the formation of liposomes, as described above as well as for the biotinylation assay.

### Quantitative determination of the biotinylated Lys (biotinylation assay)

The determination of the biotinyl groups present on the FhuA Δ1-159 Exp and FhuA Δ1-159 protein has been performed using the Invitrogen FluoReporter^® ^Biotin Quantitation Assay Kit specifically developed for proteins. Fluorescence was detected by a Tecan Spectrofluorometer (Infinite^® ^1000, Tecan Group Ltd., Männedorf Switzerland).

## Competing interests

The authors declare that they have no competing interests.

## Authors' contributions

MK and TD carried out design and performed study, data analysis and drafting of the manuscript.

MF designed research.

All author's read and approved the final manuscript.

## Supplementary Material

Additional file 1 **• Liposome DLS Data**. •  FhuA Δ1-159 Exp estimated increase in diameter • HRP Assay • Biotinylation Assay • DNA sequence of FhuA Δ1-159 Exp. Click here for file

Additional file 2 **• CD data and CONTIN deconvolution output for FhuA Δ1-159 Exp**. Click here for file
