# Supplementary material for: Engineering of an E. coli outer membrane protein FhuA with increased channel diameter
Source: J Nanobiotechnology. 2011 Aug 19;9:33. doi: 10.1186/1477-3155-9-33 (PMC3170585; doi:10.1186/1477-3155-9-33)
Supplement: Additional file 1 — • Liposome DLS Data. • FhuA Δ1-159 Exp estimated increase in diameter • HRP Assay • Biotinylation Assay • DNA sequence of FhuA Δ1-159 Exp. [file 1477-3155-9-33-S1.DOC]

**Additional File 1**

**Engineering of an *E. coli* Outer Membrane Protein FhuA with Increased Channel Diameter**

Manuel Krewinkel^1^, Tamara Dworeck^1^ and Marco Fioroni^1, §^

^1^Department of Biotechnology (Biology VI), RWTH Aachen University, Worringerweg 1, 52074 Aachen, Germany

^§^Corresponding author

Email addresses:

MK: [m.krewinkel@biotec.rwth-aachen.de](mailto:m.krewinkel@biotec.rwth-aachen.de)

TD: [t.dworeck@biotec.rwth-aachen.de](mailto:t.dworeck@biotec.rwth-aachen.de)

MF: [m.fioroni@biotec.rwth-aachen.de](mailto:m.fioroni@biotec.rwth-aachen.de)

Actual Address MF and TD:

Konrad-Müller Str. 17, 52249, Eschweiler, Germany

Content

Liposome DLS Data **S03**

FhuA ∆1-159 Exp estimated increase in diameter **S04**

HRP Assay **S04**

Biotinylation Assay **S05**

DNA sequence of FhuA ∆1-159 Exp **S07**

**DLS Measurements**

Quasielastic light scattering with a laser particle sizer (Malvern Z-sizer Nano ZS, Malvern, UK) was used to analyse the size distribution of liposomes encapsulating HRP and liposomes encapsulating HRP and accommodating FhuA Δ1-159 (Figure S2). Furthermore DLS (dynamic light scattering) was used to detect aggregate formation in FhuA Δ1-159 Δsignal solution after dialysis against buffer containing the diblock copolymer PE-PEG and PE-PEG solution without channel protein (Figure S3). The laser pinhole (Helium-Neon gas, λ= 633 nm has a 100 µm diameter with a cell width of 1 cm (disposable PS cuvettes) was based on a sample volume of 750 μL. The scattering angle was set at 173° and determined at a running T = 25 °C, equilibrated for 120 sec. 3 measurements for each sample were conducted and for each single measurement 5 runs were performed. Each single run was accumulated for 120 sec. Solutions were measured with filtration (800 µm pore size) and distributions calculated according to the CONTIN algorithm

**Figure S1. Average liposome size distribution.** Upper panel: liposomes encapsulating HRP (170.7 nm; 98 %). Lower panel: liposomes encapsulating HRP and accommodating FhuA Δ1-159 channel (161.5 nm; 97.2 %).


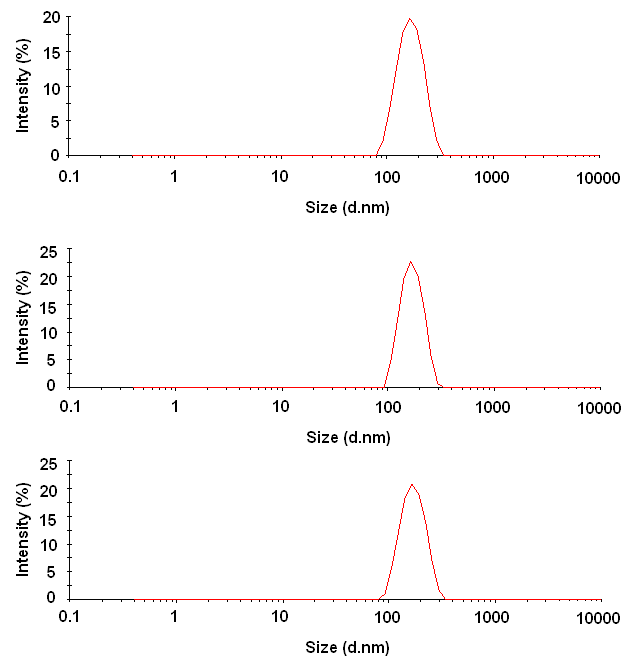


**FhuA ∆1-159 Exp estimated increase in diameter and surface area**

**Table S1**. Geometrical characteristics of the b-barrel proteins assuming a regular polygonal cross section.

| **No. of sides**  **(n)** | **No. β-sheets** | **Apothem**  **(a)** | **Radius**  **(r)** | **Channel diameter [nm]** | **Area**  **(A)** | **Protein** |
| --- | --- | --- | --- | --- | --- | --- |
| **4** | 8 | 0,500 | 0,707 | 1,673 | 1 | OmpA |
| **5** | 10 | 0,688 | 0,851 | 2,013 | 1,721 | OmpT |
| **6** | 12 | 0,866 | 1,000 | 2,367 | 2,598 |  |
| **7** | 14 | 1,038 | 1,152 | 2,727 | 3,634 |  |
| **8** | 16 | 1,207 | 1,307 | 3,092 | 4,829 | OmpF |
| **9** | 18 | 1,374 | 1,462 | 3,460 | 6,182 |  |
| **10** | 20 | 1,539 | 1,618 | 3,829 | 7,694 |  |
| **11** | 22 | 1,703 | 1,775 | 4,200 | 9,366 | FhuA |
| **12** | 24 | 1,866 | 1,932 | 4,572 | 11,196 | FhuA Δ1-159 Exp |
| **13** | 26 | 2,029 | 2,089 | 4,944 | 13,186 |  |
| **14** | 28 | 2,191 | 2,2470 | 5,318 | 15,335 |  |

As an approximation, proteins were assumed to have a simple polygonal geometry.

The side length **s is set to 1** and constant for all the polygons being the **representation of two anti-parallel β-strands.**

Polygon area A is calculated by the relation:

A = 0,5 n s a

where n = number of sides, s = side length and a is the apothem.

The apothem is calculated by the relation:

a = 0,5 s tan (n-2/2n)π 

and the radius r is calculated by the relation:

r = s / (2 sin(π/n))

All the numbers a, r and A are relative to a value of s = 1 while the channel diameter was derived from the FhuA Δ1-159 reference.

From the Table data it is possible to deduce that the FhuA Δ1-159 Exp area is ~16 % higher than the original FhuA Δ1-159 area.

**HRP Assay**

The TMB/HRP assay was carried out with HRP-loaded liposomes as a negative control (figure S2, squares) and HRP-loaded liposomes harbouring FhuA Δ1-159 Exp (figure S2, crosses).

**Figure S2. Time derivative of the absorbance was used to calculate the TMB conversion by the Lambert-Beer law.** A linear regression using “least square” method was performed to find the best linear section in the steepest region (straight lines) for HRP loaded liposomes (squares), HRP loaded liposomes with reconstituted FhuA Δ1-159 Exp (crosses) and HRP loaded liposomes with label blocked FhuA Δ1-159 Exp (triangles).


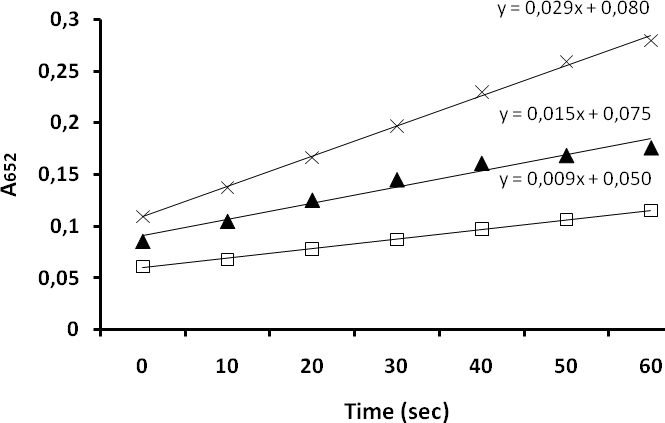


Lambert–Beer law is the empirical relationship that relates the absorption of light to the properties of the material through which the light is travelling was used to calculate the average TMB conversion [1]: A = εcL where A is the light absorbance, ε is the molar extinction coefficient of the substance (extinction coefficient of TMB oxidation product: 3.9x10^-4^ M^-1^ cm^-1^), c is the molar concentration of the substance and L is the distance that the light travels through the material [1] (see the manuscript, Table 1).

**Biotinylation Assay**

Considering that FhuA ∆1-159 Exp contains a total of 31 Lys, the expected concentration of biotin used to label these residues can be calculated as follows:

1) Protein concentration: **180 mg/ L**

2) Protein molecular weight: **66300 g/ Mol**

3) Calculated molarity of FhuA ∆1-159 Exp: **2.7 µM**

4) Calculated molar concentration of FhuA ∆1-159 Exp (100 μl of 1:2 diluted sample): **135 pmol**

**5) Expected biotin concentration in case all 31 Lys are labelled:**  **4185 pmol/ 100 µl**

Prior to the determination of biotin amount, the labelled, FhuA ∆1-159 Exp (free protein, not inserted into liposomes) was digested by proteases to reveal all biotin moieties. The relative fluorescence of the 1:100 diluted sample (total volume 50 µl) was 700 corresponding to a concentration of ~4000 pmol/ 100 µl (see calibration curve and related equation in Fig. S3).

**Figure S3.** Biotin-Assay calibration curve.


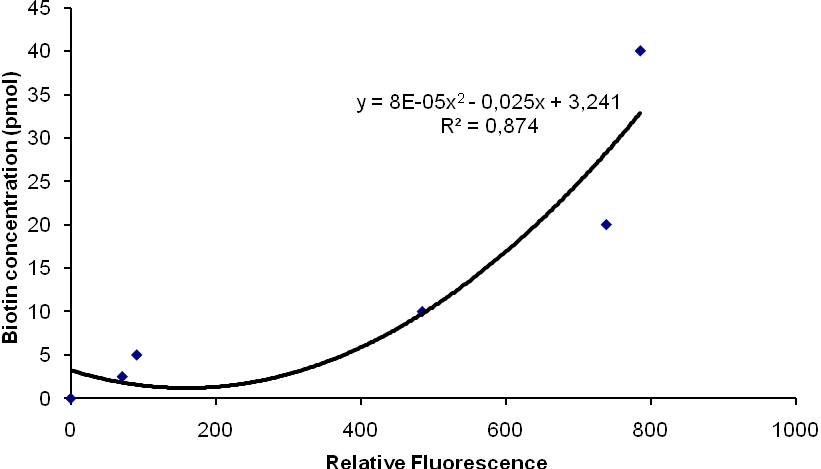


# DNA sequence of FhuA ∆1-159 Exp

The duplicated sequence is underlined in yellow.

GAATTCATGGCGCGTTCCAAAACTGCTCAGCCAAAACACTCACTGCGTAAAATCGCAGTTGTAGTAGCCACAGCGGTTAGCGGCATGTCTGTTTATGCACAGGCACTCAAAGAAGTACAGTTTAAAGCCGGCACCGACAGCCTCTTCCAGACCGGCTTTGATTTCTCCGACAGCCTGGACGACGACGGTGTGTACCTGAAAGAAGTTCAGTTTAAAGCCGGTACTGACAGCCTGTTCCAGACTGGTTTTGACTTTAGCGATTCGTTGGATGATGACGGTGTTTACTCTTATCGCCTGACCGGTCTTGCGCGTTCTGCCAATGCCCAGCAGAAAGGGTCAGAAGAGCAGCGTTATGCTATTGCACCGGCGTTCACCTGGCGTCCGGATGATAAAACCAATTTTACCTTCCTTTCTTACTTCCAGAACGAGCCGGAAACCGGTTATTACGGCTGGTTGCCGAAAGAGGGAACCGTTGAGCCGCTGCCGAACGGTAAGCGTCTGCCGACAGACTTTAATGAAGGGGCGAAGAACAACACCTATTCTCGTAATGAGAAGATGGTCGGCTACAGCTTCGATCACGAATTTAACGACACCTTTACTGTGCGTCAGAACCTGCGCTTTGCTGAAAACAAAACCTCGCAAAACAGCGTTTATGGTTACGGCGTCTGCTCCGATCCGGCGAATGCTTACAGCAAACAGTGTGCGGCATTAGCGCCAGCGGATAAAGGCCATTATCTGGCACGTAAATACGTCGTTGATGATGAGAAGCTGCAAAACTTCTCCGTTGATACCCAGTTGCAGAGCAAGTTTGCCACTGGCGATATCGACCACACCCTGCTGACCGGTGTCGACTTTATGCGTATGCGTAATGACATCAACGCCTGGTTTGGTTACGACGACTCTGTGCCACTGCTCAATCTGTACAATCCGTCTTCTCACCACCACCACCACCACGGTTCTTCTGTGAATACCGATTTCGACTTCAATGCCAAAGATCCGGCAAACTCCGGCCCTTACCGCATTCTGAATAAACAGAAACAAACGGGCGTTTATGTTCAGGATCAGGCGCAGTGGGATAAAGTGCTGGTCACCCTAGGCGGTCGTTATGACTGGGCAGATCAAGAATCTCTTAACCGCGTTGCCGGGACGACCGATAAACGTGATGACAAACAGTTTACCTGGCGTGGTGGTGTTAACTACCTGTTTGATAATGGTGTAACACCTTACTTCAGCTATAGCGAATCGTTTGAACCTTCTTCGCAAGTTGGGAAGGATGGTAATATTTTCGCACCGTCTAAAGGTAAGCAGTATGAAGTCGGCGTGAAATATGTACCGGAAGATCGTCCGATTGTAGTTACTGGTGCCGTGTATAATCTCACTAAAACCAACAACCTGATGGCGGACCCTGAGGGTTCCTTCTTCTCGGTTGAAGGTGGCGAGATCCGCGCACGTGGCGTAGAAATCGAAGCGAAAGCGGCGCTGTCGGCGAGTGTTAACGTAGTCGGTTCTTATACTTACACCGATGCGGAATACACCACCGATACTACCTATAAAGGCAATACGCCTGCACAGGTGCCAAAACACATGGCTTCGTTGTGGGCTGACTACACCTTCTTTGACGGTCCGCTTTCAGGTCTGACGCTGGGCACCGGTGGTCGTTATACTGGCTCCAGTTATGGTGATCCGGCTAACTCCTTTAAAGTGGGAAGTTATACGGTCGTGGATGCGTTAGTACGTTATGATCTGGCGCGAGTCGGCATGGCTGGCTCCAACGTGGCGCTGCATGTTAACAACCTGTTCGATCGTGAATACGTCGCCAGCTGCTTTAACACTTATGGCTGCTTCTGGGGCGCAGAACGTCAGGTCGTTGCAACCGCAACCTTCCGTTTCTGATAACTCGAG
