# Supplementary material for: Engineering of an E. coli outer membrane protein FhuA with increased channel diameter
Source: J Nanobiotechnology. 2011 Aug 19;9:33. doi: 10.1186/1477-3155-9-33 (PMC3170585; doi:10.1186/1477-3155-9-33)
Supplement: Additional file 2 — • CD data and CONTIN deconvolution output for FhuA Δ1-159 Exp. [file 1477-3155-9-33-S2.PDF]

```
 CONTIN - VERSION 2DP (MAR 1984) ( CD-1  PACK)       TEST DATA SET 1 - FOR CD PACKAGE


 REFERENCES - S.W. PROVENCHER (1982) COMPUT. PHYS. COMMUN., VOL. 27, PAGES 213-227, 229-242.
                              (1984) EMBL TECHNICAL REPORT DA07 (EUROPEAN MOLECULAR BIOLOGY
LABORATORY, HEIDELBERG, F.R. OF GERMANY)


                    INPUT DATA FOR CHANGES TO COMMON VARIABLES

 IFORMY    0    0.00000E+00
 (7F9.0)

 LAST      0    1.00000E+00

 IWT       0    5.00000E+00

 IUSER    14    3.10000E+01

 IUSER    15   -1.00000E+00

 RUSER    14    1.00000E+00

 RUSER    15    3.00000E-02

 RUSER    16    5.00000E+02

 END       0    0.00000E+00
 NSTEND   51    2.40000E+02    1.90000E+02



                                           FINAL VALUES OF CONTROL VARIABLES
 DFMIN  = 3.00000E+00
 SRMIN  = 1.00000E-02
 ALPST  = 0.00000E+00 0.00000E+00
 GMNMX  = 1.00000E+00 1.60000E+01
 PLEVEL = 5.00000E-01 5.00000E-01 5.00000E-01 5.00000E-01
 RSVMNX = 1.00000E+00 1.00000E+00 0.00000E+00 0.00000E+00
 RUSER  = 0.00000E+00 0.00000E+00 0.00000E+00 0.00000E+00 0.00000E+00 0.00000E+00
0.00000E+00 0.00000E+00 0.00000E+00 0.00000E+00
          0.00000E+00 0.00000E+00 0.00000E+00 1.00000E+00 3.00000E-02 5.00000E+02
0.00000E+00 0.00000E+00 0.00000E+00 0.00000E+00
          0.00000E+00 0.00000E+00 0.00000E+00 0.00000E+00 0.00000E+00 0.00000E+00
0.00000E+00 0.00000E+00 0.00000E+00 0.00000E+00
          0.00000E+00 0.00000E+00 0.00000E+00 0.00000E+00 0.00000E+00 0.00000E+00
0.00000E+00 0.00000E+00 0.00000E+00 0.00000E+00
          0.00000E+00 0.00000E+00 0.00000E+00 0.00000E+00 0.00000E+00 0.00000E+00
0.00000E+00 0.00000E+00 0.00000E+00 0.00000E+00
          0.00000E+00 0.00000E+00 0.00000E+00 0.00000E+00 0.00000E+00 0.00000E+00
0.00000E+00 0.00000E+00 0.00000E+00 0.00000E+00
          0.00000E+00 0.00000E+00 0.00000E+00 0.00000E+00 0.00000E+00 0.00000E+00
0.00000E+00 0.00000E+00 0.00000E+00 0.00000E+00
          0.00000E+00 0.00000E+00 0.00000E+00 0.00000E+00 0.00000E+00 0.00000E+00
0.00000E+00 0.00000E+00 0.00000E+00 0.00000E+00
          0.00000E+00 0.00000E+00 0.00000E+00 0.00000E+00 0.00000E+00 0.00000E+00
0.00000E+00 0.00000E+00 0.00000E+00 0.00000E+00
          0.00000E+00 0.00000E+00 0.00000E+00 0.00000E+00 0.00000E+00 0.00000E+00
0.00000E+00 0.00000E+00 0.00000E+00 0.00000E+00
 IGRID  =          1
 IQUAD  =          1
 IUNIT  =         -1
 IWT    =          5
 LINEPG =         60
 MIOERR =          5
 MPKMOM =          0
 MQPITR =         35
 NEQ    =          0
 NERFIT =          0
 NG     =         16
 NINTT  =          1
 NLINF  =          0
```

```
 NORDER =           -1
 ICRIT  =            1           1
 IFORMT = (5E15.6)
 IFORMW = (5E15.6)
 IFORMY = (7F9.0)
 IPLFIT =            2           2
 IPLRES =            2           2
 IPRINT =            2           3
 IUSER  =            0           0           0           0           0           0
0           0           0           0
                     0           0           0          31          -1           4
7           0           0           0
                     0           0           0           0           0           0
0           0           0           0
                     0           0           0           0           0           0
0           0           0           0
                     0           0           0           0           0           0
0           0           0           0
 IUSROU =            3           3
 LSIGN  =            0           0           0           0           0           0
0           0           0           0
                     0           0           0           0           0           0
 MOMNMX =            0           0
 NENDZ  =            0           0
 NFLAT  =            0           0           0           0           0           0
0           0
 NNSGN  =            0           0
 NQPROG =            6           6
 NSGN   =            0           0           0           0
 DOCHOS =            T
 DOMOM  =            F
 DOUSIN =            T
 DOUSNQ =            T
 LAST   =            T
 NEWPG1 =            F
 NONNEG =            F
 ONLY1  =            T
 PRWT   =            T
 PRY    =            T
 SIMULA =            F
 LUSER  =            F           F           F           F           F           F
F           F           F           F
                     F           F           F           F           F           F
F           F           F           F
                     F           F           F           F           F           F
F           F           F           F



            T            Y            T            Y            T            Y            T
          Y            T            Y
    2.400E+02  0.00000E+00    2.390E+02  6.30000E+01    2.380E+02 -1.75000E+02    2.370E+02
-2.63000E+02    2.360E+02 -3.40000E+02
    2.350E+02 -5.28000E+02    2.340E+02 -8.27000E+02    2.330E+02 -8.17000E+02    2.320E+02
-1.06700E+03    2.310E+02 -1.43200E+03
    2.300E+02 -1.73300E+03    2.290E+02 -1.97800E+03    2.280E+02 -2.23500E+03    2.270E+02
-2.37100E+03    2.260E+02 -2.66700E+03
    2.250E+02 -2.90900E+03    2.240E+02 -3.08100E+03    2.230E+02 -3.03300E+03    2.220E+02
-3.30200E+03    2.210E+02 -3.56000E+03
    2.200E+02 -3.47800E+03    2.190E+02 -3.78000E+03    2.180E+02 -3.68100E+03    2.170E+02
-3.74900E+03    2.160E+02 -3.61300E+03
    2.150E+02 -3.68600E+03    2.140E+02 -3.69000E+03    2.130E+02 -3.83600E+03    2.120E+02
-3.94500E+03    2.110E+02 -4.08800E+03
    2.100E+02 -3.90300E+03    2.090E+02 -4.04300E+03    2.080E+02 -4.17800E+03    2.070E+02
-3.89000E+03    2.060E+02 -3.70500E+03
    2.050E+02 -3.38600E+03    2.040E+02 -2.77300E+03    2.030E+02 -2.23300E+03    2.020E+02
-1.32600E+03    2.010E+02 -5.53000E+02
    2.000E+02  4.85000E+02    1.990E+02  1.08200E+03    1.980E+02  1.66200E+03    1.970E+02
3.09900E+03    1.960E+02  4.36500E+03
    1.950E+02  4.83200E+03    1.940E+02  5.08100E+03    1.930E+02  6.33200E+03    1.920E+02
6.29000E+03    1.910E+02  5.88100E+03
    1.900E+02  5.63100E+03    0.000E+00  1.00000E+00
```

0PRECIS = 1.49E-15 SRANGE = 1.00E+35 RANGE = 1.00E+35

GRID POINT	MIN IN MATRIX A	AT T =	MAX IN MATRIX A	AT T =	SCALE FACTOR
1.0000E+00	-2.4876E+04	2.22E+02	5.5079E+04	1.92E+02	2.078E-06
2.0000E+00	-1.3387E+04	2.08E+02	1.7434E+04	1.92E+02	2.078E-06
3.0000E+00	-1.1109E+04	2.11E+02	1.6667E+04	0.00E+00	2.078E-06
4.0000E+00	-1.2593E+04	2.09E+02	1.6667E+04	0.00E+00	2.078E-06
5.0000E+00	-1.3227E+04	2.22E+02	2.4142E+04	1.93E+02	2.078E-06
6.0000E+00	-9.7762E+03	2.03E+02	1.6667E+04	0.00E+00	2.078E-06
7.0000E+00	-6.6914E+03	2.24E+02	1.6667E+04	0.00E+00	2.078E-06
8.0000E+00	-1.2254E+04	2.23E+02	1.6667E+04	0.00E+00	2.078E-06
9.0000E+00	-1.2473E+04	1.99E+02	1.6667E+04	0.00E+00	2.078E-06
1.0000E+01	-1.1208E+04	2.09E+02	1.6667E+04	0.00E+00	2.078E-06
1.1000E+01	-1.3714E+04	2.09E+02	2.5943E+04	1.96E+02	2.078E-06
1.2000E+01	-1.4514E+04	2.22E+02	1.9732E+04	1.95E+02	2.078E-06
1.3000E+01	-9.9252E+03	2.12E+02	1.6667E+04	0.00E+00	2.078E-06
1.4000E+01	-1.8628E+04	2.03E+02	1.6667E+04	0.00E+00	2.078E-06
1.5000E+01	-1.4012E+04	2.10E+02	2.8231E+04	1.92E+02	2.078E-06
1.6000E+01	-1.1083E+04	2.11E+02	1.6667E+04	0.00E+00	2.078E-06

0SCALE FACTOR FOR ALPHA = 7.700E+06

0 UNREGULARIZED VARIABLES

SINGULAR VALUES

```
    4.036E-02    1.461E-02    6.268E-03    3.163E-03    1.902E-03    1.817E-03    1.383E-03
  5.122E-04    4.872E-04    3.099E-04
    2.796E-04    1.190E-04    8.184E-05    6.539E-05    2.937E-05    2.580E-05
```

1

TEST DATA SET 1 - FOR CD PACKAGE
PRELIMINARY UNWEIGHTED ANALYSIS

```
      ALPHA    ALPHA/S(1)     OBJ. FCTN.       VARIANCE      STD. DEV.    DEG FREEDOM
PROB1 TO REJECT    PROB2 TO REJECT
 * 6.01E-17      1.49E-15    1.09006E+06    1.09006E+06      1.740E+02         16.000
      0.000             1.000
```

	HELIX	BETA-SHEET	REMAINDER	SCALE FACTOR
FRACTION	0.09	0.67	0.24	0.999
STANDARD ERROR	4.5E-02	4.4E-02	7.7E-02	

0(FOR ALPHA/S(1) = 1.49E-15) PRUNS = 0.7619 PUNCOR = 0.0770 0.0002 0.0127 0.3671
0.0103

TEST DATA SET 1 - FOR CD PACKAGE
PRELIMINARY UNWEIGHTED ANALYSIS

```
      ALPHA    ALPHA/S(1)     OBJ. FCTN.       VARIANCE      STD. DEV.    DEG FREEDOM
PROB1 TO REJECT    PROB2 TO REJECT
 * 4.41E-14      1.09E-12    1.09006E+06    1.09006E+06      1.740E+02         16.000
      0.000             1.000
```

	HELIX	BETA-SHEET	REMAINDER	SCALE FACTOR
FRACTION	0.09	0.67	0.24	0.999
STANDARD ERROR	4.5E-02	4.4E-02	7.7E-02	

0(FOR ALPHA/S(1) = 1.09E-12) PRUNS = 0.7619 PUNCOR = 0.0770 0.0002 0.0127 0.3671
0.0103

TEST DATA SET 1 - FOR CD PACKAGE
PRELIMINARY UNWEIGHTED ANALYSIS

```
      ALPHA    ALPHA/S(1)     OBJ. FCTN.       VARIANCE      STD. DEV.    DEG FREEDOM
PROB1 TO REJECT    PROB2 TO REJECT
 * 3.23E-11      8.02E-10    1.09006E+06    1.09006E+06      1.740E+02         16.000
      0.000             1.000
```

	HELIX	BETA-SHEET	REMAINDER	SCALE FACTOR
FRACTION	0.09	0.67	0.24	0.999
STANDARD ERROR	4.5E-02	4.4E-02	7.7E-02	

```
0(FOR ALPHA/S(1) = 8.02E-10) PRUNS = 0.7619         PUNCOR =  0.0770  0.0002  0.0127  0.3671
 0.0103



           TEST DATA SET 1 - FOR CD PACKAGE
       PRELIMINARY UNWEIGHTED ANALYSIS

      ALPHA    ALPHA/S(1)     OBJ. FCTN.       VARIANCE      STD. DEV.    DEG FREEDOM
PROB1 TO REJECT    PROB2 TO REJECT
   2.37E-08      5.88E-07    1.09006E+06    1.09006E+06      1.740E+02         16.000
      0.000             1.000

                         HELIX   BETA-SHEET    REMAINDER                SCALE FACTOR
 FRACTION                 0.09         0.67         0.24                       0.999
 STANDARD ERROR       4.5E-02      4.4E-02      7.7E-02
0(FOR ALPHA/S(1) = 5.88E-07) PRUNS = 0.7619         PUNCOR =  0.0770  0.0002  0.0127  0.3671
 0.0103



           TEST DATA SET 1 - FOR CD PACKAGE
       PRELIMINARY UNWEIGHTED ANALYSIS

      ALPHA    ALPHA/S(1)     OBJ. FCTN.       VARIANCE      STD. DEV.    DEG FREEDOM
PROB1 TO REJECT    PROB2 TO REJECT
   1.74E-05      4.31E-04    1.25607E+06    1.11035E+06      1.739E+02         15.288
      0.000             0.694

                         HELIX   BETA-SHEET    REMAINDER                SCALE FACTOR
 FRACTION                 0.10         0.68         0.22                       0.999
 STANDARD ERROR       3.7E-02      3.6E-02      6.4E-02
0(FOR ALPHA/S(1) = 4.31E-04) PRUNS = 0.5598         PUNCOR =  0.2030  0.0005  0.0336  0.3862
 0.0034



           TEST DATA SET 1 - FOR CD PACKAGE
       PRELIMINARY UNWEIGHTED ANALYSIS

      ALPHA    ALPHA/S(1)     OBJ. FCTN.       VARIANCE      STD. DEV.    DEG FREEDOM
PROB1 TO REJECT    PROB2 TO REJECT
   1.28E-02      3.16E-01    2.66881E+08    1.24425E+08      1.574E+03          1.786
      1.000             1.000

                         HELIX   BETA-SHEET    REMAINDER                SCALE FACTOR
 FRACTION                 0.27         0.29         0.44                       0.569
 STANDARD ERROR       1.3E-02      1.1E-02      1.7E-02
0(FOR ALPHA/S(1) = 3.16E-01) PRUNS =-1.0000         PUNCOR =  0.0000  0.0004  0.0005  0.0030
 0.3683



           TEST DATA SET 1 - FOR CD PACKAGE
       PRELIMINARY UNWEIGHTED ANALYSIS

      ALPHA    ALPHA/S(1)     OBJ. FCTN.       VARIANCE      STD. DEV.    DEG FREEDOM
PROB1 TO REJECT    PROB2 TO REJECT
   4.47E-05      1.11E-03    1.82837E+06    1.31197E+06      1.854E+02         13.818
      0.048             0.958

                         HELIX   BETA-SHEET    REMAINDER                SCALE FACTOR
 FRACTION                 0.11         0.67         0.23                       0.997
 STANDARD ERROR       2.4E-02      2.6E-02      4.5E-02
0(FOR ALPHA/S(1) = 1.11E-03) PRUNS = 0.1854         PUNCOR =  0.8350  0.0068  0.3893  0.6244
 0.0008



           TEST DATA SET 1 - FOR CD PACKAGE
       PRELIMINARY UNWEIGHTED ANALYSIS
```

```
      ALPHA    ALPHA/S(1)     OBJ. FCTN.       VARIANCE      STD. DEV.    DEG FREEDOM
PROB1 TO REJECT    PROB2 TO REJECT
   1.15E-04      2.84E-03    3.45961E+06    2.11532E+06      2.295E+02         11.832
      0.969              1.000

                       HELIX   BETA-SHEET    REMAINDER                SCALE FACTOR
 FRACTION               0.08         0.61         0.31                      0.991
 STANDARD ERROR      1.4E-02      2.0E-02      2.5E-02
0(FOR ALPHA/S(1) = 2.84E-03) PRUNS = 0.0055         PUNCOR =  0.0523  0.1009  0.5092  0.8426
 0.0015



          TEST DATA SET 1 - FOR CD PACKAGE
       PRELIMINARY UNWEIGHTED ANALYSIS

      ALPHA    ALPHA/S(1)     OBJ. FCTN.       VARIANCE      STD. DEV.    DEG FREEDOM
PROB1 TO REJECT    PROB2 TO REJECT
   2.94E-04      7.29E-03    8.06379E+06    3.99736E+06      3.073E+02          9.659
      1.000              1.000

                       HELIX   BETA-SHEET    REMAINDER                SCALE FACTOR
 FRACTION               0.05         0.58         0.37                      0.972
 STANDARD ERROR      9.2E-03      2.1E-02      1.8E-02
0(FOR ALPHA/S(1) = 7.29E-03) PRUNS = 0.0590         PUNCOR =  0.0029  0.9377  0.8773  0.7843
 0.0002



          TEST DATA SET 1 - FOR CD PACKAGE
       PRELIMINARY UNWEIGHTED ANALYSIS

      ALPHA    ALPHA/S(1)     OBJ. FCTN.       VARIANCE      STD. DEV.    DEG FREEDOM
PROB1 TO REJECT    PROB2 TO REJECT
   7.55E-04      1.87E-02    2.30797E+07    9.07446E+06      4.507E+02          7.336
      1.000              1.000

                       HELIX   BETA-SHEET    REMAINDER                SCALE FACTOR
 FRACTION               0.04         0.55         0.41                      0.906
 STANDARD ERROR      8.5E-03      2.2E-02      1.7E-02
0(FOR ALPHA/S(1) = 1.87E-02) PRUNS = 0.0095         PUNCOR =  0.0065  0.1880  0.2021  0.3313
 0.0062



          TEST DATA SET 1 - FOR CD PACKAGE
       PRELIMINARY UNWEIGHTED ANALYSIS

      ALPHA    ALPHA/S(1)     OBJ. FCTN.       VARIANCE      STD. DEV.    DEG FREEDOM
PROB1 TO REJECT    PROB2 TO REJECT
   1.94E-03      4.80E-02    6.61764E+07    3.65367E+07      8.826E+02          5.094
      1.000              1.000

                       HELIX   BETA-SHEET    REMAINDER                SCALE FACTOR
 FRACTION               0.11         0.47         0.43                      0.719
 STANDARD ERROR      1.2E-02      2.9E-02      2.7E-02
0(FOR ALPHA/S(1) = 4.80E-02) PRUNS =-1.0000         PUNCOR =  0.0231  0.0166  0.0008  0.0004
 0.9751



          TEST DATA SET 1 - FOR CD PACKAGE
       PRELIMINARY UNWEIGHTED ANALYSIS

      ALPHA    ALPHA/S(1)     OBJ. FCTN.       VARIANCE      STD. DEV.    DEG FREEDOM
PROB1 TO REJECT    PROB2 TO REJECT
   4.97E-03      1.23E-01    1.25233E+08    8.80614E+07      1.342E+03          3.128
      1.000              1.000

                       HELIX   BETA-SHEET    REMAINDER                SCALE FACTOR
 FRACTION               0.22         0.34         0.44                      0.558
 STANDARD ERROR      1.4E-02      2.2E-02      2.9E-02
```

```
0(FOR ALPHA/S(1) = 1.23E-01) PRUNS =-1.0000         PUNCOR =  0.0003  0.0011  0.0002  0.0008
 0.6419
1CONTIN 2DP (MAR 84) ( CD-1 )    TEST DATA SET 1 - FOR CD PACKAGE
                       CHOSEN SOLUTION

 WEIGHTED RESIDUALS (ALPHA/S(1)= 1.11E-03) MAX=U= 4.6E+02  MIN=L=-4.8E+02 (PRUNS= 0.1854)
PUNCOR= 0.8350 0.0068 0.3893 0.6244 0.0008

 U---------+---------+---------+---------+-------*-+--
                                              *

                   *         *        *           *
   *     *                  * * *       *
      *   *    *    * *                *  *
 0*--*-*--------***--------*---*-----*-----*-*-------*
    *      * **           *      *                  *
        *   *        *  *                *     *
                       *           **              *
                         *
                                  *
                                            *

 L---------+---------+---------+---------+------*--+--
                     20                  40                  60                  80
       100                 120


0PLOT OF DATA (O) AND FIT TO DATA (X).  ORDINATES LISTED ARE FIT VALUES.

    ORDINATE  ABSCISSA
  -4.605E+01  2.40E+02                                          XO

  -6.096E+01  2.39E+02                                          XO

  -1.401E+02  2.38E+02                                         *

  -2.651E+02  2.37E+02                                        *

  -4.138E+02  2.36E+02                                       *

  -5.730E+02  2.35E+02                                     *

  -7.487E+02  2.34E+02                                  OX

  -9.423E+02  2.33E+02                                 XO

  -1.150E+03  2.32E+02                               XO

  -1.378E+03  2.31E+02                            OX

  -1.637E+03  2.30E+02                         OX

  -1.920E+03  2.29E+02                      OX

  -2.189E+03  2.28E+02                    *

  -2.460E+03  2.27E+02                 XO

  -2.696E+03  2.26E+02               *

  -2.908E+03  2.25E+02             *

  -3.101E+03  2.24E+02           *

  -3.235E+03  2.23E+02         X O

  -3.357E+03  2.22E+02        XO

  -3.480E+03  2.21E+02      OX
```

```
 -3.558E+03  2.20E+02      XO
 -3.601E+03  2.19E+02    OX
 -3.588E+03  2.18E+02     OX
 -3.549E+03  2.17E+02    O X
 -3.595E+03  2.16E+02     OX
 -3.724E+03  2.15E+02    XO
 -3.858E+03  2.14E+02   X O
 -4.024E+03  2.13E+02 X O
 -4.101E+03  2.12E+02X O
 -4.089E+03  2.11E+02*
 -4.054E+03  2.10E+02 XO
 -4.010E+03  2.09E+02 *
 -3.871E+03  2.08E+02O  X
 -3.742E+03  2.07E+02  O X
 -3.567E+03  2.06E+02    O X
 -3.394E+03  2.05E+02        *
 -2.989E+03  2.04E+02            X O
 -2.332E+03  2.03E+02                   XO
 -1.442E+03  2.02E+02                            XO
 -4.427E+02  2.01E+02                                     OX
  4.166E+02  2.00E+02                                              XO
  1.088E+03  1.99E+02                                                    *
  1.995E+03  1.98E+02                                                          O   X
  3.048E+03  1.97E+02
  XO
  4.025E+03  1.96E+02
           X  O
  4.914E+03  1.95E+02
                   OX
  5.565E+03  1.94E+02
                      O    X
  5.869E+03  1.93E+02
                            X   O
  6.093E+03  1.92E+02
                              X O
  6.055E+03  1.91E+02
                            O X
  5.682E+03  1.90E+02
                         OX
  9.970E-01  0.00E+00                                           *
```

RMS RESIDUAL FOR PTS. 1 TO 31 =   1.03E+02
RMS RESIDUAL FOR REMAINING PTS. = 2.21E+02

ERRFIT = 0.00E+00

SQUARE ROOTS OF LEAST SQUARES WEIGHTS

```
    9.6692E-03   9.6692E-03   9.6692E-03   9.6692E-03   9.6692E-03   9.6692E-03   9.6692E-03
  9.6692E-03   9.6692E-03   9.6692E-03
    9.6692E-03   9.6692E-03   9.6692E-03   9.6692E-03   9.6692E-03   9.6692E-03   9.6692E-03
  9.6692E-03   9.6692E-03   9.6692E-03
    9.6692E-03   9.6692E-03   9.6692E-03   9.6692E-03   9.6692E-03   9.6692E-03   9.6692E-03
  9.6692E-03   9.6692E-03   9.6692E-03
    9.6692E-03   4.5225E-03   4.5225E-03   4.5225E-03   4.5225E-03   4.5225E-03   4.5225E-03
  4.5225E-03   4.5225E-03   4.5225E-03
    4.5225E-03   4.5225E-03   4.5225E-03   4.5225E-03   4.5225E-03   4.5225E-03   4.5225E-03
  4.5225E-03   4.5225E-03   4.5225E-03
    4.5225E-03   3.3333E+01


   GRID POINT     MIN IN MATRIX A    AT T =     MAX IN MATRIX A    AT T =     SCALE FACTOR
   1.0000E+00         -2.4053E+02  2.22E+02          2.4909E+02  1.92E+02         2.958E-04
   2.0000E+00         -1.2449E+02  2.10E+02          7.8843E+01  1.92E+02         2.958E-04
   3.0000E+00         -1.0741E+02  2.11E+02          3.3333E+01  0.00E+00         2.958E-04
   4.0000E+00         -1.2171E+02  2.10E+02          6.6936E+01  1.90E+02         2.958E-04
   5.0000E+00         -1.2789E+02  2.22E+02          1.0918E+02  1.93E+02         2.958E-04
   6.0000E+00         -7.0295E+01  2.10E+02          3.3333E+01  0.00E+00         2.958E-04
   7.0000E+00         -6.4701E+01  2.24E+02          5.4248E+01  1.97E+02         2.958E-04
   8.0000E+00         -1.1848E+02  2.23E+02          5.8613E+01  1.96E+02         2.958E-04
   9.0000E+00         -6.9503E+01  2.10E+02          3.3333E+01  0.00E+00         2.958E-04
   1.0000E+01         -1.0708E+02  2.10E+02          5.6035E+01  1.90E+02         2.958E-04
   1.1000E+01         -1.3127E+02  2.10E+02          1.1733E+02  1.96E+02         2.958E-04
   1.2000E+01         -1.4034E+02  2.22E+02          8.9235E+01  1.95E+02         2.958E-04
   1.3000E+01         -9.5969E+01  2.12E+02          6.6382E+01  1.98E+02         2.958E-04
   1.4000E+01         -1.2146E+02  2.10E+02          3.3333E+01  0.00E+00         2.958E-04
   1.5000E+01         -1.3548E+02  2.10E+02          1.2767E+02  1.92E+02         2.958E-04
   1.6000E+01         -1.0717E+02  2.11E+02          3.3333E+01  0.00E+00         2.958E-04
0SCALE FACTOR FOR ALPHA =  5.409E+04

   0 UNREGULARIZED VARIABLES

 SINGULAR VALUES
     4.130E-02    1.018E-02    4.116E-03    3.097E-03    1.954E-03    1.507E-03    7.028E-04
   5.202E-04    4.293E-04    2.744E-04
     2.246E-04    1.180E-04    7.509E-05    4.513E-05    3.248E-05    2.414E-05
1
           TEST DATA SET 1 - FOR CD PACKAGE

      ALPHA    ALPHA/S(1)     OBJ. FCTN.       VARIANCE      STD. DEV.    DEG FREEDOM
PROB1 TO REJECT    PROB2 TO REJECT
 * 6.15E-17      1.49E-15    3.66052E+01    3.66052E+01      1.008E+00         16.000
      0.000              1.000

    ORDINATE    ERROR  ABSCISSA
   1.026E+00  2.1E-01  1.00E+00
      ......X......
  -2.561E-01  2.4E-01  2.00E+00                            .......X.......

   6.680E-01  5.2E-01  3.00E+00
................X................
  -4.400E-01  8.5E-02  4.00E+00                           ..X...

  -1.422E+00  4.8E-01  5.00E+00X.............

   4.343E-01  1.4E-01  6.00E+00                                                    ....X....

   7.491E-01  1.4E-01  7.00E+00
.....X....
   3.895E-01  1.5E-01  8.00E+00                                                  .....X....

   1.866E+00  1.9E-01  9.00E+00
                                .....X
  -1.406E+00  3.6E-01  1.00E+01X..........

  -3.643E-01  7.6E-02  1.10E+01                             ...X..

   3.460E-01  1.4E-01  1.20E+01                                                 ....X.....

   8.212E-02  1.2E-01  1.30E+01                                          ...X....
```

```
  -1.666E-01  1.3E-01  1.40E+01                                  ....X....

   3.764E-01  1.6E-01  1.50E+01                                                 .....X.....

  -8.879E-01  5.3E-01  1.60E+01................X................


                       HELIX   BETA-SHEET    REMAINDER                SCALE FACTOR
 FRACTION               0.08         0.66         0.26                       0.995
 STANDARD ERROR      4.1E-02      4.7E-02      7.4E-02
0(FOR ALPHA/S(1) = 1.49E-15) PRUNS = 0.7727         PUNCOR =  0.1428  0.0014  0.2139  0.4502
 0.0716



              TEST DATA SET 1 - FOR CD PACKAGE

      ALPHA    ALPHA/S(1)     OBJ. FCTN.       VARIANCE      STD. DEV.    DEG FREEDOM
PROB1 TO REJECT    PROB2 TO REJECT
 * 4.51E-14      1.09E-12    3.66052E+01    3.66052E+01      1.008E+00         16.000
      0.000              1.000

    ORDINATE    ERROR  ABSCISSA
   1.026E+00  2.1E-01  1.00E+00
      ......X......
  -2.561E-01  2.4E-01  2.00E+00                            .......X.......

   6.680E-01  5.2E-01  3.00E+00
................X................
  -4.400E-01  8.5E-02  4.00E+00                           ..X...

  -1.422E+00  4.8E-01  5.00E+00X.............

   4.343E-01  1.4E-01  6.00E+00                                                    ....X....

   7.491E-01  1.4E-01  7.00E+00
.....X....
   3.895E-01  1.5E-01  8.00E+00                                                  .....X....

   1.866E+00  1.9E-01  9.00E+00
                                .....X
  -1.406E+00  3.6E-01  1.00E+01X...........

  -3.643E-01  7.6E-02  1.10E+01                             ...X..

   3.460E-01  1.4E-01  1.20E+01                                                 ....X.....

   8.212E-02  1.2E-01  1.30E+01                                          ...X....

  -1.666E-01  1.3E-01  1.40E+01                                  ....X....

   3.764E-01  1.6E-01  1.50E+01                                                 .....X.....

  -8.879E-01  5.3E-01  1.60E+01................X................


                       HELIX   BETA-SHEET    REMAINDER                SCALE FACTOR
 FRACTION               0.08         0.66         0.26                       0.995
 STANDARD ERROR      4.1E-02      4.7E-02      7.4E-02
0(FOR ALPHA/S(1) = 1.09E-12) PRUNS = 0.7727         PUNCOR =  0.1428  0.0014  0.2139  0.4502
 0.0716



              TEST DATA SET 1 - FOR CD PACKAGE

      ALPHA    ALPHA/S(1)     OBJ. FCTN.       VARIANCE      STD. DEV.    DEG FREEDOM
PROB1 TO REJECT    PROB2 TO REJECT
 * 3.31E-11      8.02E-10    3.66052E+01    3.66052E+01      1.008E+00         16.000
      0.000              1.000
```

```
    ORDINATE    ERROR  ABSCISSA
   1.026E+00  2.1E-01  1.00E+00
      ......X......
  -2.561E-01  2.4E-01  2.00E+00                              .......X.......

   6.680E-01  5.2E-01  3.00E+00
................X................
  -4.400E-01  8.5E-02  4.00E+00                             ..X...

  -1.422E+00  4.8E-01  5.00E+00X.............

   4.343E-01  1.4E-01  6.00E+00                                                    ....X....

   7.491E-01  1.4E-01  7.00E+00
.....X....
   3.895E-01  1.5E-01  8.00E+00                                                  .....X....

   1.866E+00  1.9E-01  9.00E+00
                                .....X
  -1.406E+00  3.6E-01  1.00E+01X..........

  -3.643E-01  7.6E-02  1.10E+01                                ...X..

   3.460E-01  1.4E-01  1.20E+01                                                 ....X.....

   8.212E-02  1.2E-01  1.30E+01                                          ...X....

  -1.666E-01  1.3E-01  1.40E+01                                  ....X....

   3.764E-01  1.6E-01  1.50E+01                                                 .....X.....

  -8.879E-01  5.3E-01  1.60E+01................X................


                       HELIX   BETA-SHEET    REMAINDER                SCALE FACTOR
 FRACTION               0.08         0.66         0.26                       0.995
 STANDARD ERROR      4.1E-02      4.7E-02      7.4E-02
0(FOR ALPHA/S(1) = 8.02E-10) PRUNS = 0.7727         PUNCOR =  0.1428  0.0014  0.2139  0.4502
 0.0716



           TEST DATA SET 1 - FOR CD PACKAGE

      ALPHA    ALPHA/S(1)     OBJ. FCTN.       VARIANCE      STD. DEV.    DEG FREEDOM
PROB1 TO REJECT    PROB2 TO REJECT
 * 2.43E-08      5.88E-07    3.66053E+01    3.66052E+01      1.008E+00         16.000
      0.000              1.000

    ORDINATE    ERROR  ABSCISSA
   1.026E+00  2.1E-01  1.00E+00
      ......X......
  -2.561E-01  2.4E-01  2.00E+00                              .......X.......

   6.680E-01  5.2E-01  3.00E+00
................X................
  -4.400E-01  8.5E-02  4.00E+00                             ..X...

  -1.422E+00  4.8E-01  5.00E+00X.............

   4.343E-01  1.4E-01  6.00E+00                                                    ....X....

   7.491E-01  1.4E-01  7.00E+00
.....X....
   3.895E-01  1.5E-01  8.00E+00                                                  .....X....

   1.866E+00  1.9E-01  9.00E+00
                                .....X
  -1.406E+00  3.6E-01  1.00E+01X..........

  -3.643E-01  7.6E-02  1.10E+01                                ...X..
```

```
   3.460E-01  1.4E-01  1.20E+01                                                ....X.....

   8.212E-02  1.2E-01  1.30E+01                                         ...X....

  -1.666E-01  1.3E-01  1.40E+01                                 ....X....

   3.764E-01  1.6E-01  1.50E+01                                                .....X.....

  -8.879E-01  5.3E-01  1.60E+01................X................


                         HELIX   BETA-SHEET    REMAINDER                SCALE FACTOR
 FRACTION                 0.08         0.66         0.26                       0.995
 STANDARD ERROR        4.1E-02      4.7E-02      7.4E-02
0(FOR ALPHA/S(1) = 5.88E-07) PRUNS = 0.7727         PUNCOR =  0.1429  0.0014  0.2139  0.4502
 0.0716



             TEST DATA SET 1 - FOR CD PACKAGE

      ALPHA    ALPHA/S(1)     OBJ. FCTN.       VARIANCE      STD. DEV.    DEG FREEDOM
PROB1 TO REJECT    PROB2 TO REJECT
   1.78E-05      4.31E-04    4.51068E+01    3.80763E+01      1.017E+00         15.192
      0.000              0.813

    ORDINATE    ERROR  ABSCISSA
   8.141E-01  1.7E-01  1.00E+00
  ......X......
  -7.659E-02  2.0E-01  2.00E+00                                .......X.......

   4.069E-01  3.4E-01  3.00E+00
............X...........
  -3.895E-01  7.1E-02  4.00E+00                         ...X..

  -1.077E+00  3.8E-01  5.00E+00....X.............

   3.318E-01  1.1E-01  6.00E+00                                                ....X....

   6.630E-01  1.1E-01  7.00E+00
...X....
   3.806E-01  1.3E-01  8.00E+00                                                 .....X....

   1.670E+00  1.6E-01  9.00E+00
                                .....X
  -1.205E+00  3.1E-01  1.00E+01X..........

  -3.004E-01  6.7E-02  1.10E+01                              ..X..

   2.199E-01  1.1E-01  1.20E+01                                            ....X....

  -3.319E-02  9.5E-02  1.30E+01                                    ...X....

  -2.219E-01  1.2E-01  1.40E+01                               ....X....

   3.226E-01  1.5E-01  1.50E+01                                               .....X.....

  -5.168E-01  3.5E-01  1.60E+01           ............X............


                         HELIX   BETA-SHEET    REMAINDER                SCALE FACTOR
 FRACTION                 0.08         0.66         0.26                       0.989
 STANDARD ERROR        3.5E-02      4.1E-02      6.4E-02
0(FOR ALPHA/S(1) = 4.31E-04) PRUNS = 0.5598         PUNCOR =  0.3632  0.0049  0.3407  0.5021
 0.0142



             TEST DATA SET 1 - FOR CD PACKAGE

      ALPHA    ALPHA/S(1)     OBJ. FCTN.       VARIANCE      STD. DEV.    DEG FREEDOM
PROB1 TO REJECT    PROB2 TO REJECT
```

```
   1.31E-02      3.16E-01    1.19287E+04    2.54701E+03      7.100E+00          1.472
      1.000              1.000

    ORDINATE    ERROR  ABSCISSA
  -2.160E-03  2.7E-03  1.00E+00X.....

   3.012E-02  1.1E-03  2.00E+00
..X..
   3.642E-02  1.6E-03  3.00E+00
         ...X...
   2.414E-02  2.2E-03  4.00E+00                                              ....X.....

   2.498E-02  1.1E-03  5.00E+00                                                  ..X..

   4.397E-02  1.6E-03  6.00E+00
                        ...X...
   4.729E-02  1.7E-03  7.00E+00
                              ...X....
   2.702E-02  1.3E-03  8.00E+00                                                     ...X...

   4.943E-02  2.1E-03  9.00E+00
                                 ....X
   2.984E-02  1.0E-03  1.00E+01
..X.
   2.907E-02  1.5E-03  1.10E+01
...X...
   1.681E-02  1.6E-03  1.20E+01                                 ...X...

   3.485E-02  1.7E-03  1.30E+01
      ...X...
   3.243E-02  2.8E-03  1.40E+01
......X.....
   2.551E-02  1.3E-03  1.50E+01                                                   ..X...

   3.605E-02  1.6E-03  1.60E+01
         ...X...

                          HELIX   BETA-SHEET    REMAINDER                SCALE FACTOR
 FRACTION                  0.27         0.29         0.44                       0.486
 STANDARD ERROR         9.4E-03      7.0E-03      1.1E-02
0(FOR ALPHA/S(1) = 3.16E-01) PRUNS =-1.0000         PUNCOR =  0.0000  0.0000  0.0000  0.0000
 0.0023



              TEST DATA SET 1 - FOR CD PACKAGE

        ALPHA    ALPHA/S(1)     OBJ. FCTN.       VARIANCE      STD. DEV.    DEG FREEDOM
PROB1 TO REJECT    PROB2 TO REJECT
   4.57E-05      1.11E-03    6.86016E+01    4.98373E+01      1.139E+00         13.555
      0.337              0.994

    ORDINATE    ERROR  ABSCISSA
   3.694E-01  9.7E-02  1.00E+00                                                  .....X.....

   1.988E-01  1.5E-01  2.00E+00                                      ........X........

   1.755E-01  1.4E-01  3.00E+00                                     ........X.......

  -2.890E-01  5.8E-02  4.00E+00                    ...X...

  -4.332E-01  1.9E-01  5.00E+00    .........X..........

   1.690E-01  8.5E-02  6.00E+00                                        ....X.....

   4.800E-01  6.8E-02  7.00E+00
...X....
   3.021E-01  9.7E-02  8.00E+00                                              .....X.....

   1.233E+00  1.2E-01  9.00E+00
                              ......X
  -6.979E-01  2.0E-01  1.00E+01X.........
```

```
  -1.621E-01  5.8E-02  1.10E+01                        ...X...

   1.260E-02  7.9E-02  1.20E+01                                ....X....

  -2.487E-01  6.3E-02  1.30E+01                    ...X...

  -3.452E-01  9.8E-02  1.40E+01             .....X.....

   2.837E-01  1.3E-01  1.50E+01                                            ......X.......

  -7.827E-02  1.4E-01  1.60E+01                        ........X.......


                       HELIX   BETA-SHEET    REMAINDER                SCALE FACTOR
 FRACTION               0.07         0.63         0.30                       0.970
 STANDARD ERROR      2.3E-02      3.4E-02      4.5E-02
0(FOR ALPHA/S(1) = 1.11E-03) PRUNS = 0.1730         PUNCOR =  0.5192  0.0607  0.9145  0.9857
 0.0008



           TEST DATA SET 1 - FOR CD PACKAGE

      ALPHA    ALPHA/S(1)     OBJ. FCTN.       VARIANCE      STD. DEV.    DEG FREEDOM
PROB1 TO REJECT    PROB2 TO REJECT
   1.17E-04      2.84E-03   1.25390E+02    7.86924E+01      1.394E+00         11.509
      0.991              1.000

    ORDINATE    ERROR  ABSCISSA
   2.041E-02  4.3E-02  1.00E+00                             ....X....

   2.867E-01  7.6E-02  2.00E+00
.......X.......
   1.390E-01  4.3E-02  3.00E+00                                        ....X....

  -1.638E-01  4.7E-02  4.00E+00           ....X.....

  -6.371E-02  5.4E-02  5.00E+00                    .....X.....

   8.861E-02  5.8E-02  6.00E+00                                  .....X......

   3.666E-01  4.1E-02  7.00E+00
....X....
   1.305E-01  6.3E-02  8.00E+00                                     ......X......

   7.240E-01  7.6E-02  9.00E+00
                                .......X
  -2.189E-01  7.4E-02  1.00E+01   .......X.......

   8.923E-03  4.9E-02  1.10E+01                           .....X....

  -1.422E-01  5.2E-02  1.20E+01            .....X.....

  -3.307E-01  4.3E-02  1.30E+01X....

  -3.263E-01  6.6E-02  1.40E+01X......

   3.277E-01  7.9E-02  1.50E+01
........X.......
   6.791E-02  4.2E-02  1.60E+01                                 ....X....


                       HELIX   BETA-SHEET    REMAINDER                SCALE FACTOR
 FRACTION               0.06         0.58         0.36                       0.915
 STANDARD ERROR      1.3E-02      3.3E-02      2.9E-02
0(FOR ALPHA/S(1) = 2.84E-03) PRUNS = 0.1637         PUNCOR =  0.1216  0.3579  0.6599  0.8271
 0.0001



           TEST DATA SET 1 - FOR CD PACKAGE
```

```
      ALPHA    ALPHA/S(1)     OBJ. FCTN.       VARIANCE      STD. DEV.    DEG FREEDOM
PROB1 TO REJECT    PROB2 TO REJECT
   3.01E-04      7.29E-03    2.66412E+02    1.58732E+02      1.926E+00          9.230
      1.000             1.000

    ORDINATE    ERROR  ABSCISSA
  -2.517E-02  2.1E-02  1.00E+00                                 ....X....

   1.622E-01  3.5E-02  2.00E+00
 ......X......
   1.217E-01  2.0E-02  3.00E+00
....X...
  -1.014E-01  3.5E-02  4.00E+00                 ......X......

   1.518E-02  2.2E-02  5.00E+00                                        ....X....

   1.199E-01  4.4E-02  6.00E+00
........X........
   2.943E-01  3.4E-02  7.00E+00
                             ......X......
   1.813E-02  4.3E-02  8.00E+00                                    ........X........

   3.292E-01  4.1E-02  9.00E+00
                                  .......X
  -5.113E-02  2.8E-02  1.00E+01                           .....X.....

   9.867E-02  4.1E-02  1.10E+01
........X.......
  -2.197E-01  3.5E-02  1.20E+01X......

  -1.903E-01  3.0E-02  1.30E+01.....X.....

  -1.264E-01  3.4E-02  1.40E+01            ......X.......

   2.418E-01  4.2E-02  1.50E+01
                 ........X.......
   8.151E-02  2.0E-02  1.60E+01                                                     ...X....


                          HELIX   BETA-SHEET    REMAINDER                SCALE FACTOR
 FRACTION                  0.06         0.55         0.38                       0.768
 STANDARD ERROR      1.3E-02      4.1E-02      3.1E-02
0(FOR ALPHA/S(1) = 7.29E-03) PRUNS = 0.0626         PUNCOR =  0.1980  0.9172  0.5711  0.5398
 0.0003



           TEST DATA SET 1 - FOR CD PACKAGE

      ALPHA    ALPHA/S(1)     OBJ. FCTN.       VARIANCE      STD. DEV.    DEG FREEDOM
PROB1 TO REJECT    PROB2 TO REJECT
   7.73E-04      1.87E-02    5.45864E+02    3.53090E+02      2.795E+00          6.787
      1.000             1.000

    ORDINATE    ERROR  ABSCISSA
   2.783E-02  1.0E-02  1.00E+00
....X...
   5.828E-02  1.6E-02  2.00E+00
   .....X.....
   9.876E-02  1.3E-02  3.00E+00
                    .....X....
  -6.463E-02  2.2E-02  4.00E+00                        .......X.......

   2.817E-02  1.2E-02  5.00E+00
....X....
   8.571E-02  2.4E-02  6.00E+00
            ........X.......
   1.461E-01  2.5E-02  7.00E+00
                                 ........X
  -2.297E-02  2.1E-02  8.00E+00                                     .......X.......
```

```
   1.261E-01  1.8E-02  9.00E+01
                         ......X......
   5.568E-03  9.7E-03  1.00E+01                                                  ...X....

   6.218E-02  2.4E-02  1.10E+01
 ........X........
  -1.523E-01  2.2E-02  1.20E+01X.......

  -5.856E-02  2.2E-02  1.30E+01                        .......X.......

  -7.016E-03  1.7E-02  1.40E+01                                              .....X......

   1.120E-01  1.6E-02  1.50E+01
                     .....X......
   8.111E-02  1.3E-02  1.60E+01
           .....X....

                       HELIX   BETA-SHEET    REMAINDER                SCALE FACTOR
 FRACTION               0.13         0.50         0.37                       0.526
 STANDARD ERROR      1.4E-02      4.4E-02      3.4E-02
0(FOR ALPHA/S(1) = 1.87E-02) PRUNS = 0.1733         PUNCOR =  0.6202  0.6363  0.0635  0.0123
 0.0847



            TEST DATA SET 1 - FOR CD PACKAGE

      ALPHA    ALPHA/S(1)     OBJ. FCTN.       VARIANCE      STD. DEV.    DEG FREEDOM
PROB1 TO REJECT    PROB2 TO REJECT
   1.98E-03      4.80E-02   1.10337E+03    6.24608E+02      3.630E+00          4.599
      1.000              1.000

    ORDINATE    ERROR  ABSCISSA
   3.837E-02  7.9E-03  1.00E+00
            .......X......
   3.081E-02  7.9E-03  2.00E+00
    .......X......
   6.470E-02  9.6E-03  3.00E+00
                              .......X
  -2.081E-02  1.1E-02  4.00E+00                       ........X.........

   2.158E-02  4.5E-03  5.00E+00
...X....
   3.702E-02  8.9E-03  6.00E+00
          .......X........
   4.259E-02  1.1E-02  7.00E+00
             .........X.........
  -1.413E-02  8.7E-03  8.00E+00                              .......X.......

   5.907E-02  5.8E-03  9.00E+00
                             .....X....
   1.519E-02  3.3E-03  1.00E+01
...X...
   3.676E-02  1.1E-02  1.10E+01
         ........X.........
  -6.086E-02  1.1E-02  1.20E+01X........

  -6.592E-03  1.3E-02  1.30E+01                                ...........X..........

   2.292E-02  7.5E-03  1.40E+01
......X......
   5.862E-02  6.1E-03  1.50E+01
                             .....X....
   5.787E-02  9.5E-03  1.60E+01
                         .......X.....

                       HELIX   BETA-SHEET    REMAINDER                SCALE FACTOR
 FRACTION               0.26         0.38         0.36                       0.383
 STANDARD ERROR      1.3E-02      2.5E-02      2.9E-02
0(FOR ALPHA/S(1) = 4.80E-02) PRUNS = 0.0002         PUNCOR =  0.0071  0.0111  0.0004  0.0001
 0.6062
```

```
           TEST DATA SET 1 - FOR CD PACKAGE

      ALPHA    ALPHA/S(1)     OBJ. FCTN.       VARIANCE      STD. DEV.    DEG FREEDOM
PROB1 TO REJECT    PROB2 TO REJECT
   5.09E-03      1.23E-01    3.01445E+03    1.09215E+03      4.707E+00          2.702
      1.000             1.000

    ORDINATE    ERROR  ABSCISSA
   1.422E-02  5.0E-03  1.00E+00                                ..........X.........

   2.886E-02  2.9E-03  2.00E+00
 .....X......
   3.850E-02  4.2E-03  3.00E+00
                 ........X........
   6.271E-03  4.9E-03  4.00E+00                 ..........X.........

   2.274E-02  1.8E-03  5.00E+00                                                    ....X...

   3.651E-02  3.3E-03  6.00E+00
               ......X......
   3.771E-02  4.8E-03  7.00E+00
              .........X.........
   9.832E-03  3.8E-03  8.00E+00                          .......X........

   4.531E-02  3.2E-03  9.00E+00
                               ......X
   2.187E-02  1.6E-03  1.00E+01                                                   ...X...

   3.016E-02  4.2E-03  1.10E+01
 ........X........
  -7.566E-03  4.4E-03  1.20E+01X........

   2.062E-02  5.1E-03  1.30E+01
..........X.........
   2.502E-02  4.4E-03  1.40E+01
........X........
   3.432E-02  2.4E-03  1.50E+01
            .....X....
   3.645E-02  4.3E-03  1.60E+01
             ........X........

                         HELIX   BETA-SHEET    REMAINDER                SCALE FACTOR
 FRACTION                 0.28         0.31         0.41                       0.401
 STANDARD ERROR      9.1E-03      1.3E-02      1.8E-02
0(FOR ALPHA/S(1) = 1.23E-01) PRUNS = 0.0000         PUNCOR =  0.0000  0.0000  0.0000  0.0000
 0.0145
1CONTIN 2DP (MAR 84) ( CD-1 )    TEST DATA SET 1 - FOR CD PACKAGE
                         CHOSEN SOLUTION

 WEIGHTED RESIDUALS (ALPHA/S(1)= 1.11E-03) MAX=U= 2.2E+00  MIN=L=-2.2E+00 (PRUNS= 0.1730)
PUNCOR= 0.5192 0.0607 0.9145 0.9857 0.0008

 U---------+---------+---------+---------+-------*-+--
                   *                          *

         *                  *         *           *
   *      *           *      *         **            *
                    *         * *
  *   *        *
 0-----*--------------------*---------*-----*--*--------
     *     *    * *       *                    *
    *        **  *      *        *       * *       *
            *        *         *                    *
        *                          **
                       *
                         *
                                  *         *
 L---------+---------+---------+---------+------*--+--
                     20                   40                  60                  80
       100                 120
```

```
0PLOT OF DATA (O) AND FIT TO DATA (X).  ORDINATES LISTED ARE FIT VALUES.

    ORDINATE  ABSCISSA
  -4.289E+01  2.40E+02                                          XO

  -4.677E+01  2.39E+02                                          XO

  -1.157E+02  2.38E+02                                         OX

  -2.328E+02  2.37E+02                                        *

  -3.772E+02  2.36E+02                                       *

  -5.391E+02  2.35E+02                                     *

  -7.264E+02  2.34E+02                                  OX

  -9.377E+02  2.33E+02                                 XO

  -1.171E+03  2.32E+02                               XO

  -1.412E+03  2.31E+02                            *

  -1.667E+03  2.30E+02                         OX

  -1.934E+03  2.29E+02                      OX

  -2.178E+03  2.28E+02                    *

  -2.426E+03  2.27E+02                  *

  -2.652E+03  2.26E+02               *

  -2.867E+03  2.25E+02             *

  -3.071E+03  2.24E+02           *

  -3.226E+03  2.23E+02         X O

  -3.364E+03  2.22E+02        XO

  -3.496E+03  2.21E+02      OX

  -3.587E+03  2.20E+02      XO

  -3.644E+03  2.19E+02    OX

  -3.633E+03  2.18E+02     *

  -3.589E+03  2.17E+02    O X

  -3.611E+03  2.16E+02     *

  -3.704E+03  2.15E+02    XO

  -3.812E+03  2.14E+02   X O

  -3.950E+03  2.13E+02  XO

  -4.015E+03  2.12E+02 XO

  -4.005E+03  2.11E+02OX

  -3.978E+03  2.10E+02  *

  -3.925E+03  2.09E+02 OX

  -3.770E+03  2.08E+02O   X
```

```
  -3.631E+03  2.07E+02  O  X

  -3.470E+03  2.06E+02    O  X

  -3.388E+03  2.05E+02        *

  -3.047E+03  2.04E+02           X  O

  -2.437E+03  2.03E+02                  X O

  -1.551E+03  2.02E+02                           X O

  -4.806E+02  2.01E+02                                     OX

   4.874E+02  2.00E+02                                                *

   1.177E+03  1.99E+02                                                      OX

   2.068E+03  1.98E+02                                                            O   X

   3.048E+03  1.97E+02
   XO
   3.972E+03  1.96E+02
             X   O
   4.881E+03  1.95E+02
                      *
   5.568E+03  1.94E+02
                         O    X
   5.852E+03  1.93E+02
                                 X   O
   6.023E+03  1.92E+02
                                  X  O
   6.000E+03  1.91E+02
                                 OX
   5.780E+03  1.90E+02
                              O X
   9.700E-01  0.00E+00                                                 *

1CONTIN VERSION 2DP (MAR 1984) ( CD-1  PACKAGE)  ++++++++++++++++++++++++++++++++  CHOSEN
SOLUTION  ++++++++++++++++++++++++++++++++

           TEST DATA SET 1 - FOR CD PACKAGE

      ALPHA    ALPHA/S(1)     OBJ. FCTN.       VARIANCE      STD. DEV.    DEG FREEDOM
PROB1 TO REJECT    PROB2 TO REJECT
   4.57E-05      1.11E-03    6.86016E+01    4.98373E+01      1.139E+00         13.555
      0.337              0.994

    ORDINATE    ERROR  ABSCISSA
   3.694E-01  9.7E-02  1.00E+00                                                    .....X.....

   1.988E-01  1.5E-01  2.00E+00                                        ........X........

   1.755E-01  1.4E-01  3.00E+00                                       ........X.......

  -2.890E-01  5.8E-02  4.00E+00                         ...X...

  -4.332E-01  1.9E-01  5.00E+00           .........X.........

   1.690E-01  8.5E-02  6.00E+00                                          ....X.....

   4.800E-01  6.8E-02  7.00E+00
...X....
   3.021E-01  9.7E-02  8.00E+00                                                 .....X.....

   1.233E+00  1.2E-01  9.00E+00
                               ......X
  -6.979E-01  2.0E-01  1.00E+01X..........

  -1.621E-01  5.8E-02  1.10E+01                               ...X...

   1.260E-02  7.9E-02  1.20E+01                                       ....X....
```

```
 -2.487E-01  6.3E-02  1.30E+01                   ...X...

 -3.452E-01  9.8E-02  1.40E+01            .....X.....

  2.837E-01  1.3E-01  1.50E+01                                                ......X.......

 -7.827E-02  1.4E-01  1.60E+01                       ........X.......
```

	HELIX	BETA-SHEET	REMAINDER	SCALE FACTOR
FRACTION	0.07	0.63	0.30	0.970
STANDARD ERROR	2.3E-02	3.4E-02	4.5E-02	
